# Genetic Correlations Between Photosynthetic and Yield Performance in Maize Are Different Under Two Heat Scenarios During Flowering

**DOI:** 10.3389/fpls.2019.00566

**Published:** 2019-04-30

**Authors:** Vlatko Galic, Mario Franic, Antun Jambrovic, Tatjana Ledencan, Andrija Brkic, Zvonimir Zdunic, Domagoj Simic

**Affiliations:** ^1^Department of Maize Breeding and Genetics, Agricultural Institute Osijek, Osijek, Croatia; ^2^Centre of Excellence for Biodiversity and Molecular Plant Breeding, Zagreb, Croatia

**Keywords:** flowering, grain yield, heat, photosynthetic performance, pleiotropy, quantitative trait loci, *Zea mays*

## Abstract

Chlorophyll fluorescence (ChlF) parameters are reliable early stress indicators in crops, but their relations with yield are still not clear. The aims of this study are to examine genetic correlations between photosynthetic performance of JIP-test during flowering and grain yield (GY) in maize grown under two heat scenarios in the field environments applying quantitative genetic analysis, and to compare efficiencies of indirect selection for GY through ChlF parameters and genomic selection for GY. The testcrosses of 221 intermated recombinant inbred lines (IRILs) of the IBM*Syn4* population were evaluated in six environments at two geographically distinctive locations in 3 years. According to day/night temperatures and vapor pressure deficit (VPD), the two locations in Croatia and Turkey may be categorized to the mild heat and moderate heat scenarios, respectively. Mild heat scenario is characterized by daytime temperatures often exceeding 33°C and night temperatures lower than 20°C while in moderate heat scenario the daytime temperatures often exceeded 33°C and night temperatures were above 20°C. The most discernible differences among the scenarios were obtained for efficiency of electron transport beyond quinone A (Q_A_) [ET/(TR-ET)], performance index on absorption basis (PI_ABS_) and GY. Under the moderate heat scenario, there were tight positive genetic correlations between ET/(TR-ET) and GY (0.73), as well as between PI_ABS_ and GY (0.59). Associations between the traits were noticeably weaker under the mild heat scenario. Analysis of quantitative trait loci (QTL) revealed several common QTLs for photosynthetic and yield performance under the moderate heat scenario corroborating pleiotropy. Although the indirect selection with ChlF parameters is less efficient than direct selection, ET/(TR-ET) and PI_ABS_ could be efficient secondary breeding traits for selection under moderate heat stress since they seem to be genetically correlated with GY in the stressed environments and not associated with yield performance under non-stressed conditions predicting GY during flowering. Indirect selection through PI_ABS_ was also shown to be more efficient than genomic selection in moderate heat scenario.

## Introduction

Crop response to different stresses affected by weather anomalies including climate change is highly complex. It involves changes at the genetic and physiological levels that facilitate avoiding and/or coping with stress. The strategy of avoiding is commonly applied in agronomy where stress can be circumvented by agricultural practice (Jung and Müller, 2009; Teixeira et al., 2013). On the other hand, modern plant breeding aims to conduct studies on trait physiology, phenotyping, and genotyping on how to cope with stress (Araus et al., 2012; Fahad et al., 2017). Drought is one of the major stressors limiting the crop production in rainfed areas, and the likelihood of observing the anomalies in seasonal precipitation is increasing worldwide (IPCC, 2014). However, Lobell et al. (2013) demonstrated that extreme heat as a stressor had more critical role for maize production than drought in the United States, corroborating previous statistical studies of rainfed maize yields showing a strong negative yield response to accumulation of extreme temperatures (>30°C) and relative weak response to seasonal rainfall. In the future, up to 10 million tons of maize may be lost in the developing world each year as temperatures increase and precipitation patterns change due to the anthropogenic emission of greenhouse gasses. This yield loss could eventually affect 140 million people.

At the physiological level, the impact of heat on the photosynthetic apparatus is considered to be of particular significance since photosynthesis is the most sensitive of all plant cell processes to high temperatures (Sharkey and Schrader, 2006). It is often inhibited before other cell functions are impaired causing changes in the reduction-oxidation properties of PSII acceptors and reduce the efficiency of photosynthetic electron transport in both Photosystem I and II (Mathur et al., 2014). The effects of heat and other stresses are manifested in the behavior of the fluorescence transients (Rohacek and Bartak, 1999; Sayed, 2003) by reducing both the ratio of reduced primary acceptors to reaction center (RC) and the ratio of reduced secondary to primary acceptors. The ChlF induction transients (O-J-I-P) can be translated via JIP-test into several phenomenological and biophysical parameters (Strasser et al., 1995, 2000, 2004) that quantify PSII functioning and can reflect the activity of the whole photosynthetic machinery (Strasser et al., 2004). One of the most often employed ChlF parameters is *F*_v_/*F*_m_, which gives the information about the proportion of the light absorbed by chlorophyll in the PSII that is used in photochemical processes. However, the intensity of *F*_v_/*F*_m_ is determined only by the changes in minimum (F_0_) and maximum (F_m_) fluorescence and has been proved to be insensitive for assessing mildly stressed plants (e.g., Van Heerden et al., 2004; Oukarroum et al., 2007). The most powerful and most comprehensive parameters are the performance indexes (PI_ABS_ and PI_Total_) taking into the account all of the main photochemical processes. PI_ABS_ appeared to be very suitable and sensitive parameter to investigate plant overall photosynthetic performance in moderately stressed environments (Živčák et al., 2008; Šimić et al., 2014). The chlorophyll *a* fluorescence parameters are being used extensively in stress physiology in a range of plant species under controlled conditions and it is also adaptable to field conditions (Šimić et al., 2014). This is particularly important for crop improvement, because stress studies conducted under controlled conditions inadequately reflect natural environmental conditions. Additionally, chlorophyll *a* fluorescence measurement is non-destructive and fast. It generates considerable amount of data belonging to high-throughput phenotyping methods.

The relations between ChlF parameters and grain yield (GY) in the field environments are not elucidated. Kalaji et al. (2017) stressed that in quite a few studies ChlF parameters were considered as selection tools in plant breeding. They emphasized the importance of obtaining ChlF-related traits showing a high correlation with yield or plant performance in addition to ChlF-related traits that are specific for tolerance/resistance to the stress of interest. We assume that the use of certain ChlF parameters of JIP-test as secondary traits for selection under moderate heat stress would be an efficient breeding strategy if ChlF parameters would be genetically variable, genetically correlated with grain yield in the target environment, and not associated with any yield loss under non-stressed conditions. Ribaut et al. (2009) had similar expectations by reviewing secondary breeding traits under drought conditions. Furthermore, any secondary trait or trait related allele can help confer stress tolerance given the precise design of the right scenario as it helps to define the breeding strategies in the modeling process (Tardieu, 2011). Millet et al. (2016) showed the allelic effects vary with temperature scenarios, the night temperatures playing the major role. Variation of adaptive traits or combination of those traits in a set of specific environmental scenarios along with its shared variability and high heritability is crucial for adaptive trait to be useful in breeding for stress tolerance (Tardieu et al., 2018).

The objectives of this study were to examine genetic correlations between photosynthetic performance of JIP-test during flowering and GY in maize grown under two heat scenarios in the field environments by applying quantitative genetic analysis and to provide the genetic information about the usability and scalability of the ChlF data in maize breeding for heat stress tolerance. Additionally, we aimed to compare the efficiencies of indirect selection for GY through ChlF parameters and genomic selection for GY.

## Materials and Methods

### Plant Materials and Field Experiments

The maize IBM*Syn4* population (Lee et al., 2002) was chosen according to the results presented by Šimić et al. (2014) for photosynthetic parameters in the population *per se* The 221 IBMS*yn4* IRILs were included in the experiment (see the list in Supplementary Tables S1 and S2). From the cross between the IBM*Syn4* IRILs and an Iodent elite inbred, property of the Agricultural Institute Osijek, 221 testcross genotypes were generated and used in this investigation.

The testcrosses were evaluated in six environments at two locations in Croatia and Turkey during three consecutive years 2014, 2015, and 2016. Each year-location combination was considered as an environment. Locations were Osijek, Croatia, 45°32′20.6″N 18°44′21.1″E (OS), and Altinova near Ayvalik, Turkey, 39°11′43″N 26°46′34″E (AY). Experimental locations OS and AY were found to match the heat scenarios defined by Millet et al. (2016). Thus, trials in location OS fell in category of *Hot(day)* scenario, while the environments in location AY were categorized as the *Hot* scenario. Characteristics of the *Hot(day)* scenario are hot daytime temperatures that often exceed 33°C, and night temperatures lower than 20°C. *Hot* scenario is characterized by the daytime temperatures that normally exceed 33°C and night (low) temperatures are above 20°C (Table 1). For brevity, we renamed the scenarios *Hot* and *Hot(day)* to mild heat and moderate heat scenarios, respectively, indicating different stress levels. Weather data for both locations was collected from the webpages of The Weather Underground (The Weather Channel Interactive, Atlanta, GA, United States) for the closest available meteorological stations to the experimental locations (18 km for AY in Ayvalik, and 9 km for OS). VPD of the air was applied to confirm the heat scenario classification. VPD was calculated following the guidelines from Allen et al. (1998) from temperature and air humidity data. Field trials consisting of the 221 IRIL testcrosses of the IBM population along with three checks were planted in randomized single row plots in two replications with 20 plants per row (plot size 7 m^2^) according to alpha-lattice design (Patterson and Williams, 1976). The experiments in all environments were planted at the end of April and harvested in the first decade of October. Usual local crop management practice for high-yielding maize was applied according to local rain-fed (OS) and watered (AY) regimes taking into account the soil characteristics and the previous cropping.

**TABLE 1:** Weather data for six environments during July in 3 years (2014–2016) in two locations Osijek, Croatia (OS) and Ayvalik, Turkey (AY).

Environment	Aver. t (°C)	24 h low t (°C)	Precip. (mm)	No. rainy days	VPD (Pa)^a^	VPD dh (Pa)^b^
OS14	22.3 ± 2.1	16.8 ± 1.9	82.6	15	807 ± 237	2185 ± 619
OS15	24.6 ± 3.2	18.0 ± 2.4	24.9	6	1272 ± 402	2945 ± 954
OS16	23.0 ± 2.7	17.3 ± 2.1	114.2	8	1041 ± 342	2299 ± 776
AY14	26.1 ± 1.8	22.7 ± 1.5	0.3	1	1821 ± 356	3517 ± 763
AY15	27.1 ± 1.5	22.2 ± 1.1	0	0	1943 ± 332	3510 ± 704
AY16	27.4 ± 1.3	23.4 ± 1.1	1	1	1935 ± 335	3643 ± 716
Mean OS${}^{c}$	23.3 ± 2.6	17.4 ± 2.1	73.9	9.7	1040${}^{b}$	2476${}^{b}$
Mean AY	26.9 ± 1.5	22.8 ± 1.2	0.4	0.7	1899${}^{a}$	3556${}^{a}$

*^a^24 h average vapor pressure deficit (VPD). ^b^Highest daily 1 h average vapor pressure deficit. ^c^Mean differences for VPD between locations were tested by Tukey HSD test. Different letters indicate significant difference at α = 0.05.*

### Chlorophyll *a* Fluorescence Measurements

Chlorophyll *a* fluorescence (ChlF) was measured by Plant Efficiency Analyzer Handy-PEA (Hansatech, United Kingdom). All the measurements occurred between 6.30 am and 9.30 am after 30 min of sample dark adaptation. Measurements were performed on attached ear leaves of four tasseling plants in the middle of each plot. We chose ear leaf as it was found that it best represents total canopy chlorophyll content (Ciganda et al., 2009). Briefly, all plant samples exhibited polyphasic ChlF rise after the dark adaptation in the first second upon illumination with high-intensity light (3500 μmol m^–2^ s^–1^) (Strasser et al., 2000, 2004). The application of the saturating light pulse (red light, wavelength peak at 650 nm) induces chlorophyll *a* fluorescence increase from minimal fluorescence (F_0_, O step) when all reaction centers (RC) are open, to maximal fluorescence (F_m_, P step) when all RC are closed. During the first 2 ms, changes were recorded every 10 ms, and every 1 ms thereafter. Data obtained were used to calculate several biophysical parameters that describe the photochemistry of PSII according to Strasser et al. (2004). Calculated parameters used in quantitative genetic analysis were: $\frac{R\,C}{A\,B\,S}=\frac{\gamma \,R\,C}{1-\gamma \,R\,C}$ – RC/ABS; $\frac{E\,T_{0}}{T\,R_{0}-E\,T_{0}}=\frac{\psi \,E_{0}}{1-\psi \,E_{0}}$ – ET/(TR-ET); $\frac{\gamma \,R\,C}{1-\gamma \,R\,C}*\frac{\varphi_{P_{0}}}{1-\varphi_{P_{0}}}*\frac{\psi \,E_{0}}{1-\psi \,E_{0}}$ – performance index (potential) for energy conservation from photons absorbed by PSII to the reduction of intersystem electron acceptors.

### Quantitative Genetic Analysis

In the statistical analysis of individual trials, genotypic variance of the 221 IRIL testcrosses and checks, as well as block, replication and error variances for the three JIP-test traits and grain yield were calculated by analysis of variance (ANOVA). Generally, there were no significant effects of replication and block. This suggests that no considerable changes occurred in weather conditions during the measurement time span (6.30–9.30 am). In the combined ANOVA, effects of environment, genotype, genotype × environment interaction, and error were estimated on adjusted entry mean values from the individual trial analyses. Heritability on a genotype (entry) mean basis (Hallauer et al., 2010) were estimated as $h^{2}=s_{G}^{2}/\left(s_{G}^{2}++s_{\mathrm{GE}}^{2}/e+s_{e}^{2}/re\right)$, where $s_{G}^{2}$ is the variance component due to genotype (IRIL testcrosses), $s_{\mathrm{GE}}^{2}$ is the variance component due to genotype × environment interaction and s^2^_e_ the pooled error variance, whereas *r* is the number of replications per environment (2) and *e* is the number of environments (8). The genetic correlations were firstly estimated from genetic covariances via multivariate analysis of variance (MANOVA – Anderson, 1958; Mode and Robinson, 1959). Analysis of variance was performed, and genetic correlation coefficients according to MANOVA were calculated using PLABSTAT program, Version 3A (Utz, 2005). Additionally, the genetic correlations between genome-wide marker effects among the traits within a scenario and the genetic correlations between the means of the two scenarios were approximated using the method outlined by Ziyomo and Bernardo (2013). Briefly, the ridge regression best linear unbiased predictions (rrBLUP) model (Endelman, 2011) was fitted with the data from two scenarios, and the marker effects were obtained. The genetic correlation between the phenotypes was calculated as Pearson’s product-moment correlation between the marker effects for two phenotypes and tested for significance.

The R/sommer package (Covarrubias-Pazaran, 2016) was used for calculation of BLUPs with genotype as random factor on scenario basis. Genotypic BLUPs for each scenario were used as the input data for QTL mapping. We used a linkage map of the IBM*Syn4* population anchored with 2178 molecular markers, mostly SNPs and SSRs (Andorf et al., 2010). Total map length was 7090 cM and the average distance between markers was 3.2 cM (Supplementary Figure S1). As IBM population consists of recombinant inbred lines developed through four successive generations of intermating Syn4 (recombination), centiMorgan of IBM population can be considered IBM centiMorgan, as there was the increase in both map size and density (Lee et al., 2002; Falque et al., 2005). QTL results reported here are in IBM cM (1 IBM cM ∼ 4 cM in F_2_) on IBM2 Neighbors map. QTL mapping procedure was conducted using inclusive composite interval mapping method (ICIM) in QTL IciMapping software, version 4.1 (Meng et al., 2015). LOD threshold to declare putative QTL significant was calculated on 1000 permutations basis. Calculated LOD thresholds in mild heat scenario were 3.48 for RC/ABS, 3.44 for ET/(TR-ET), 3.58 for PI_ABS_ and 3.60 for grain yield. In moderate heat scenario, the thresholds were 3.51 for RC/ABS, 3.73 for ET/(TR-ET), 3.54 for PI_ABS_ and 3.68 for grain yield. The largest *P*-value for entering variables into the stepwise regression was set to p=0.001. Step size for the analysis was set to 1 cM. Confidence intervals of the detected QTL were determined based on one-LOD unit drop in both forward and backward directions. Since a common QTL for ET/(TR-ET) and grain yield was detected, we performed a joint QTL analysis for better understanding of the relationships between the traits. The maximum LOD score along the interval was taken as the position of the QTL. MCIM likelihood ratio test was used from joint analysis for BLUPs of ET/(TR-ET) and grain yield averaged across the environments, to determine if QTL detected by MCIM had pleiotropic effects. JZmapQTL procedure of the Windows QTL Cartographer software version 2.5 (Wang et al., 2011) was used for detecting pleiotropy. The LOD significance threshold for joint mapping was 4.72 generated by permutation analysis (α = 0.05, experiment wide).

### Indirect Selection Efficiency and Genomewide Marker Effects

To test the usability of the ChlF parameters genetically correlated with yield for the indirect selection in testcrosses, the efficiency of indirect selection over direct phenotypic selection was calculated according to Bernardo (2010) as $\left|r_{g}\right|\,h_{x}^{2}/h_{y}^{2}$ where | $r_{g}|$ is the absolute value of genetic correlation, $h_{x}^{2}$ is heritability of secondary trait, and $h_{y}^{2}$ is the heritability of the trait of interest. Furthermore, we aimed to compare the efficiency of the indirect selection through the ChlF parameters with the efficiency of genomic selection. The genome-wide marker effects were calculated using the rrBLUP method as implemented in the R/rrBLUP mixed model solver with markers as random effects (Endelman, 2011). The rrBLUP model was chosen as it outperforms other models for genomic predictions in case of a large number of small-effect QTLs affecting the trait (Heffner et al., 2009; Kwong et al., 2017). The cross-validation procedure was run with 500 random folds of the 80% of individuals that comprised the training set used to calculate the marker effects. The rest of the set (20% of individuals) was used to obtain the genomic predictions (validation set). The accuracy of genomic predictions ($r_{M\,P}$) was calculated in each of 500 cycles as correlation between observed and predicted values. The efficiency of the genomic selection was calculated according to Dekkers (2007) and Ziyomo and Bernardo (2013) as $r_{M\,P}/h^{2}$. The means of the 500 random folds were tested for significance of the differences from zero by the means of one sample *t*-test in R.

## Results

Weather conditions differed substantially between Osijek, Croatia (OS) and Ayvalik, Turkey (AY) (Table 1). During the three growing seasons in 2014, 2015, 2016, temperatures were consistently and significantly higher in AY than those in OS. In average, temperatures were higher in AY for 3.6°C in July. As expected, the differences in precipitation were even more obvious. VPD values (both 24 h average VPD and highest daily 1 h average VPD) varied considerably between OS and AY, and respective mean VPD values were significantly higher in AY (1899 Pa) than those in OS (1040 Pa) according to the Tukey HSD test.

Means for the three ChlF parameters measured during flowering on ear-leaf as well as GY (t/ha) in testcrosses of 221 IRILs of the IBM*Syn4* maize population across the six environments were presented in Supplementary Tables S1, S2. Parameters RC/ABS, ET/(TR-ET) and PI_ABS_ as well as GY varied significantly either across the environments or between the two locations (Supplementary Table S3). The most discernable differences among environments and locations were obtained for ET/(TR-ET) (1.91–2.60), PI_ABS_ (3.14–5.21) and GY (5.76–12.15 t/ha). Considerably higher mean values were observed in OS environments for ET/(TR-ET) (2.39 in OS, 2.08 in AY) PI_ABS_ (4.70 in OS and 3.94 in AY) and grain yield (10.95 in OS and 8.33 in AY). In average, mean values for all ChlF traits and GY were significantly higher in OS than in AY. Variance components for RC/ABS, ET/(TR-ET), PI_ABS_ and grain yield were presented in Table 2 showing the greatest variance components due to environment (E), followed by the variance component due to genotype (G) and genotype × environment interaction (GE) whereby the GE interaction was not significant across the traits. Heritability estimates were the highest for PI_ABS_ (72.7%) and GY (70.8%).

**TABLE 2:** Estimated variance components and heritabilities along with their standard errors for three JIP test parameters and grain yield in 221 testcrosses of the IBM IRILs combined over six environments.

Variance component	RC/ABS^a^	ET/(TR-ET)	PI_ABS_	Yield
Genotype (G)	$0.0002\pm 0.0001^{**}$	$0.0049\pm 0.0022^{**}$	$0.1371\pm 0.0255^{**}$	$0.68\pm 0.13^{**}$
Environment (E)	$0.0008\pm 0.0004^{**}$	$0.0619\pm 0.0334^{**}$	$0.5173\pm 0.2784^{**}$	$4.94\pm 2.65^{**}$
Genotype × environment (GE)	-0.0002±0.0001	-0.0061±0.0059	-0.1365±0.0457	-0.71±0.24
Residual	0.0024±0.0001	0.1095±0.004	0.8903±0.0325	4.77±0.17
Heritability (%)	54.6±6.7	37.7±8.1	72.7±5.0	70.8±5.0

**F-test of corresponding mean squares significant at the 0.01 probability level. ^a^RC/ABS, reaction centers involved in $Q_{A}^{-}$ reduction per Photosystem II antenna chlorophyll; ET/(TR-ET), electron transport beyond $Q_{A}^{-}$; PI_ABS_, performance index (potential) for energy conservation from photons absorbed by PSII to the reduction of intersystem electron acceptors.

As expected, the tightest genetic correlations estimated both from genetic covariances and from genome-wide marker effects were between PI_ABS_ and its components RC/ABS and ET/(TR-ET) in both scenarios (Table 3). Generally, the genetic correlations between all traits were considerably stronger under moderate heat scenario. The only exception was the genetic correlation estimated from genetic covariances between ET/(TR-ET) and PI_ABS_. Genetic correlations from genetic covariances in mild heat scenario ranged from −0.32 between ET/(TR-ET) and GY to 0.82 between ET/(TR-ET) and PI_ABS_, while genotypic correlations estimated from genome-wide marker effects ranged from −0.06 between PI_ABS_ and GY to 0.65 between ET/(TR-ET) and PI_ABS_. In moderate heat scenario, the correlations estimated from genetic covariances ranged from 0.51 between RC/ABS and GY to 0.92 between RC/ABS and PI_ABS_, while correlations from genome-wide marker effects were detected in range from 0.37 between ET/(TR-ET) and GY to 0.85 between RC/ABS and PI_ABS_. Notably, the direction of genetic correlations between some ChlF parameters and GY, have changed from negative to positive and became stronger and significant under moderate heat scenario. Generally, the correlations estimated from genetic covariances agreed well with the correlations from genome-wide marker effects. The strongest correlations from genome-wide marker effects between scenarios were for PI_ABS_, followed by GY.

**TABLE 3:** Genetic correlations estimated from genetic variances and covariances (above the diagonal) and genetic correlations based on genome-wide marker effects (below the diagonal) for three JIP-test parameters and grain yield in the field experiments classified to two heat scenarios (*mild heat* and *moderate heat*) over 3 years in 221 testcrosses of the IBM IRILs.

Scenario	Trait^a^	RC/ABS^a^	ET/(TR-ET)	PI_ABS_	Yield
Mild heat	RC/ABS		0.31	0.67++	0.09
	ET/(TR-ET)	$0.40^{**}$		0.82++	-0.32
	PI_ABS_	$0.60^{**}$	$0.65^{**}$		-0.17
	Yield	-0.05	0.02	-0.06	
Moderate heat	RC/ABS		0.59++	0.92++	0.51
	ET/(TR-ET)	$0.67^{**}$		0.70++	0.73++
	PI_ABS_	$0.85^{**}$	$0.79^{**}$		0.59++
	Yield	$0.38^{**}$	$0.37^{**}$	$0.47^{**}$	
Between scenarios		$0.22^{**}$	0.03	$0.49^{**}$	$0.39^{**}$

Genetic correlations based on genome-wide marker effects were estimated also between scenarios for a specific trait. ^a^RC/ABS, reaction centers involved in $Q_{A}^{-}$ reduction per Photosystem II antenna chlorophyll; ET/(TR-ET), electron transport beyond $Q_{A}^{-}$; PI_ABS_, performance index (potential) for energy conservation from photons absorbed by PSII to the reduction of intersystem electron acceptors. ++, The values are larger than two times the standard error. **Significance at *P* = 0.01.

Inclusive composite interval QTL mapping for four ChlF parameters and grain yield revealed five and six significant QTLs under mild heat and moderate heat scenarios, respectively (Table 4). The highest LOD score was detected for a QTL for RC/ABS under mild heat scenario. There were no common QTLs across the two heat scenarios. However, two pairs of QTLs were collocated under moderate heat scenario: QTLs for ET/(TR-ET) and grain yield on Chromosome 1, and QTLs for RC/ABS and PI_ABS_ on Chromosome 10. The QTL for ET/(TR-ET) explained the greatest percentage of phenotypic variation (8.27%). Almost all QTLs (except one for yield) had negative signs of the additive effect means. The direction of allelic effects means that almost all favorable alleles came from IBM population parental line B73.

**TABLE 4:** Results of inclusive composite interval mapping (ICIM) in BLUPs for each heat scenario.

Scenario	Trait^a^	Chr.	Position	Left marker	Right marker	LOD	PVE (%)^b^	Add.^c^
Mild heat	RC/ABS	2	118	AY109516	dmt102b	3.87	5.46	-0.002
	RC/ABS	4	748	cat3	umc1707	4.96	7.00	-0.002
	RC/ABS	9	238	asg63a	umc2340	5.29	7.32	-0.002
	PI_ABS_	2	72	bnlg1017	umc1980	3.63	6.66	-0.041
	Yield	8	490	psy2	AY110539	3.71	7.88	0.132
Moderate heat	RC/ABS	4	160	umc2280	AY110573	3.63	5.96	-0.003
	RC/ABS	10	483	npi254b	bnlg1450	3.52	5.59	-0.003
	ET/(TR-ET)	1	583	asg16b	mmp123	4.58	8.27	-0.018
	PI_ABS_	4	160	umc2280	AY110573	4.05	6.58	-0.061
	PI_ABS_	10	483	npi254b	bnlg1450	3.86	6.04	-0.059
	Yield	1	583	asg16b	mmp123	4.21	5.77	-0.216

*LOD threshold was 3.51 calculated on 1000 permutations basis at the significance level of α=0.0.5. ^a^RC/ABS, reaction centers involved in $Q_{A}^{-}$ reduction per Photosystem II antenna chlorophyll; ET/(TR-ET), electron transport beyond $Q_{A}^{-}$; PI_ABS_, performance index (potential) for energy conservation from photons absorbed by PSII to the reduction of intersystem electron acceptors. ^b^Phenotypic variation explained by the QTL. ^c^Additive effect-negative sign of the effect means that the favorable allele comes from B73.*

**FIGURE 1:**
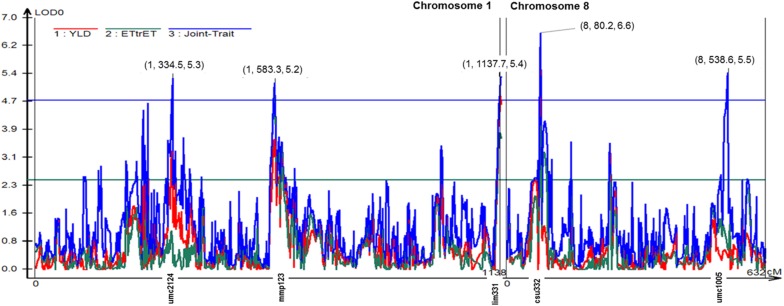
Likelihood odds ratio (LOD0) profile from multitrait composite interval mapping (MCIM) for grain yield (yld) and the chlorophyll fluorescence parameter ET/(TR-ET) (electron transport beyond $Q_{A}^{-}$) mapped on chromosomes 1 and 8. The solid blue line indicates the MCIM LOD significance threshold of 4.72 for joint mapping generated by permutation analysis (α = 0.05, experiment wide). The numbers above the five peaks indicate (chromosome number, position in cM, the LOD score). Marker name associated with the respective peak was given along the *x*-axis.

The joint multi-trait composite interval mapping (MCIM) for ET/(TR-ET) and grain yield revealed other four significant QTLs on Chromosomes 1 and 8 according to MCIM likelihood ratio test (Figure 1). There were totally three peaks on Chromosome 1 and two peaks on Chromosome 8 that exceeded the threshold of LOD = 4.72. The highest peak was on Chromosome 8, position 80.2 reaching LOD score of 6.6.

**FIGURE 2:**
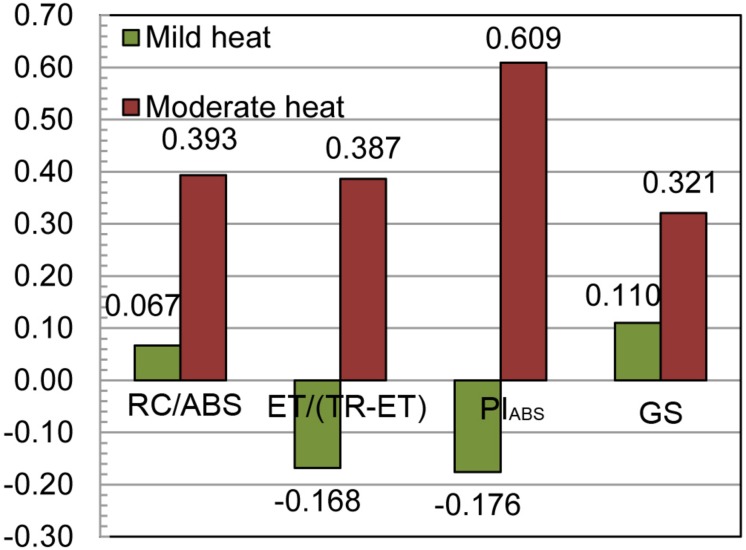
The relative efficiencies of the indirect selection for grain yield through chlorophyll *a* fluorescence parameters RC/ABS (reaction centers involved in $Q_{A}^{-}$ reduction per Photosystem II antenna chlorophyll), ET/(TR-ET) (electron transport beyond $Q_{A}^{-}$) and PI_ABS_ [performance index (potential) for energy conservation from photons absorbed by PSII to the reduction of intersystem electron acceptors] and genomic selection (GS) in two heat scenarios.

The calculated efficiency of indirect selection for grain yield through ChlF parameters was lower than the efficiency of direct selection in both heat scenarios, ranging from −17.6 to 6.7% in mild heat and 38.7 to 60.9% in moderate heat scenario (Figure 2). The obtained efficiencies of genomic selection were also low at 11.0 and 32.1% of the efficiency of the direct selection for grain yield in mild and moderate heat scenarios, respectively. Remarkably, the highest efficiency of indirect selection was through PI_ABS_ in moderate heat scenario.

## Discussion

Assigning individual field experiments to scenarios based on environmental conditions can be used for assessing the performance of genotypes and the contribution of genomic regions (QTLs) under current and future abiotic stress situations (Millet et al., 2016). On the other hand, a thorough understanding of physiological responses of plants to stress is imperative for developing crop plants with improved stress tolerance. In the context of heat stress, bringing together genetic and ecophysiological analyses should accelerate characterization and improvement of crop thermotolerance in breeding programs. VPD as a function of air moisture and temperature defines the difference between saturated air vapor pressure and actual saturation for a given temperature (Allen et al., 1998). Effects of VPD are manifold, but the most prominent is the effect on transpiration rate by inducing the decrease in stomatal conductance (Gholipoor et al., 2013) and increase in evaporative demand. Beside the components of photosystem are sensitive to heat stress, stomatal closure results in limitation of CO_2_ transfer to the sites of carboxylation in chloroplast stroma, directly limiting the photosynthetic rate (Perdomo et al., 2016). Alterations in various photosynthetic attributes is one of the physiological responses to heat stress (Wahid et al., 2007) where applying ChlF parameters plays important role in quantifying the alterations. The scenarios *Hot* and *Hot(day)* classified by Millet et al. (2016) are equivalent to the mild heat and moderate heat scenarios in our study additionally differentiated by different average *F*_v_/*F*_m_ values during flowering. Thus, a heat scenario in our study was characterized by different daytime and night temperature, VPD and *F*_v_/*F*_m_ during flowering evidenced by significantly higher mean values for all presented quantitative traits under mild heat conditions.

Although ChlF induction transients (O-J-I-P) can be translated via JIP-test into numerous phenomenological and biophysical parameters, just a few ChlF parameters are actual traits suitable for appropriate and worthwhile quantitative genetic analysis. Furthermore, apart from having inadequate statistical properties (*F*_v_/*F*_m_ and TR/DI), they may not be satisfactorily genetically variable (RC/ABS) or may not have high heritability (ET/(TR-ET)). On the other hand, PI_ABS_ as a multivariate expression is sufficiently genetically variable and it may have higher heritability than GY. Along with positive and tight genetic correlations with GY in stress environments, it indicates that indirect selection for heat tolerance through PI_ABS_ is feasible.

Another way to improve the effectiveness of selection for thermotolerance is to select for molecular markers associated with performance traits under heat stress via QTL analysis. Our previous studies on ChlF parameters under moderate drought stress in maize inbred lines B73, Mo17 as well as their IRILs of the IBMSyn4 population demonstrated that they act as an excellent resource for physiological, genetic and genomic studies of photosynthetic alterations (Lepeduš et al., 2012; Šimić et al., 2014). Apart from detecting putative QTLs for thermotolerance, the premise in this study was using the linkage map of the IBM*Syn4* population anchored with 2178 molecular markers in order to (i) compare genetic correlations from genetic covariance with genetic correlations from genome-wide marker effects, (ii) confirm genetic correlations through finding some common genes underlying the phenotypic variation in multiple traits, and (iii) compare efficiencies of indirect selection for GY through ChlF parameters and genomic selection for GY. Firstly, genetic correlations from genetic covariance agreed well with genetic correlations from genome-wide marker effects yielding similar results. Equivalent outcome for genetic correlations for drought tolerance in the IBMSyn4 population was reported by Ziyomo and Bernardo (2013).

By detecting putative QTLs, we restricted the choice of candidates to structural genes, which could be directly related to the examined traits in activating stress responsive mechanisms. The data about markers in the marker intervals of detected QTLs and co−localized putative candidate genes with their respective positions are publicly available in IBM 2008 Neighbors Map via Maize Genetics and Genomic Database at http://www.maizegdb.org (Schaeffer et al., 2008). According to this genetic map based on the IBM population, although there are several genes within the QTL intervals, only few or one of them are genes with known or assumed function and may be involved in the process of photosynthesis.

In the *mild heat* scenario QTL for RC/ABS on chromosome 4 was mapped close to gene *cat3* coding for enzyme catalase. Catalase is one of the most important antioxidant enzymes related to maintaining the cell and cellular compartment redox balance. Catalase can also affect the process of photosynthesis (Mhamdi et al., 2012). Within the QTL interval for PI_ABS_ on chromosome 2, position 72 cM, gene *ereb197* coding for AP2-EREBP-transcription factor is found. AP2/EREBP are known to be involved in regulation of transcription in various abiotic stresses in many plant species (Dietz et al., 2010), and PI_ABS_ is a sensitive indicator of many abiotic stress conditions (Oukarroum et al., 2007; Živčák et al., 2008; Stefanov et al., 2011; Lepeduš et al., 2012; Šimić et al., 2014). On chromosome 8, position 490 cM, the position of detected QTL overlapped with the position of the gene *psy2* whose product is the enzyme phytoene synthase 2. Phytoene synthase gene family is essential for photosynthesis and photoprotection in plants and can influence the grain yield (Li et al., 2008).

The QTL detected on chromosome 1, position 483 cM for RC/ABS and PI_ABS_ in *Moderate heat* scenario is located in the region where the gene *sig2A* (sigma factor *sig2A*) is found. The role of plant sigma factors is the regulation of the expression of plastid genome (Lysenko, 2007) and they appear to be up-regulated by light. Another QTL for both RC/ABS and PI_ABS_ in *Moderate heat* scenario was detected on chromosome 10, position 403 cM. Within the QTL interval, transcription factor *Zmsbp28* is found, belonging to the group of SQUAMOSA promoter binding protein-like (SPL) transcription factors (TFs). SPL TFs play a crucial role in maize growth and development but are also involved in plant response on abiotic stresses (Mao et al., 2016). Chao et al. (2017) found that SPL TFs in *Arabidopsis* can confer tolerance to high temperatures during the reproductive stages of development. Measurements of the ChlF in this current research were performed during the flowering, and the mentioned QTL were detected in *moderate heat* scenario.

We found that a region on a chromosome 1, position 583 cM holds a pleiotropic loci or two tightly linked genomic regions controlling grain yield and ChlF parameter ET/(TR-ET) also confirmed by MCIM procedure in Windows QTL Cartographer. In the proximity (6 IBM cM, 1.5 cM) from the peak of the QTL, gene *zmm6* is found coding for MADS6, a well-known promoter of TPP (trehalose-phosphate-phosphatase) included in regulation of plant carbon budget and sugar signaling in response to the abiotic stresses (Griffiths et al., 2016). MADS6 plays an essential role in endosperm nutrient accumulation in reproductive organs, balancing the source-sink relations in maize. TPP product trehalose-6-phosphate (T6P) is tightly linked to regulation of plant free sucrose levels, and increased levels of T6P might be ensuring optimal gene expression for biosynthetic processes. Nuccio et al. (2015) overexpressed the gene encoding TPP in maize. The overexpression resulted in 31–123% increase in yield in water-stressed environments, and 9–49% in non-drought environments. It was found that overexpression led to reduction of T6P levels, and increase in levels of sucrose in ear spikelets. Increased levels of sucrose might also help maintain photosynthetic processes during stress.

MCIM procedure of Windows QTL Cartographer revealed four more pleiotropic loci for ET/(TR-ET) and Yield on chromosomes 1 and 8 (Figure 1). Pleiotropic QTL on chromosome 1, position 334.5 cM was detected in the genomic region near the gene *sod4* coding for cytosolic superoxide dismutase 4 enzyme. Reactive oxygen species (ROS) are known to be generated as a consequence of biotic and abiotic stresses, and can impair photosystem (Gill and Tuteja, 2010). Expression of *sod4* might have influenced the traits by alleviation of oxidative stress damage. On the end of chromosome 1, position 1137.7 cM, *ZmAKINβγ1* gene is found coding for AKINβγ protein kinase. AKINβγ kinase is localized in cytosol and chloroplasts, and its activation is closely related to stress signaling and starch granule formation (Avila-Castañeda et al., 2014). Starch formation is a key process in maize yield formation and balancing the production of sucrose plays vital role for plants when carbon is sparse. AKINβγ seems to be one of the crucial enzymes in diurnal change and response of plants to daytime osmotic changes (Pokhilko and Ebenhöh, 2015). The QTL on chromosome 8, position 80.2 cM was detected in proximity of two tightly linked genes that might have influenced each individual trait, grain yield and ET/(TR-ET). The gene *mem1* coding for MEM1 (Mesophyll Expression Module 1) is a *cis*-element in regulation of C_4_ carbonic anhydrase, an essential enzyme in function of maize photosynthetic apparatus. MEM 1 mediates the transport of carbon to photosynthesizing cells and is important in abiotic stress conditions for replenishment of cells with carbon dioxide (Kaul et al., 2011). Other gene found in the proximity of the detected QTL was *sod3b* coding for antioxidant enzyme superoxide-dismutase 3B. Another QTL detected on chromosome 8, position 538.6 cM was located near the gene *cyc4* coding for cyclin-4, regulator of cyclin-dependent kinases involved in active progression of cell cycle (Buendía-Monreal et al., 2011) and regulation of growth in abiotic stress conditions (Aslam et al., 2015). Due to the canonical role of cyclins, and their regulatory role in whole plant growth and development, this gene might have had the effect on both traits. The detected loci were found in the gene rich regions and indicated the pleiotropy that possibly caused the genetic correlations between the ChlF traits and grain yield, although the amount of the genetic variance explained by the detected QTLs was generally low. QTL analysis captures only phenotypic variation of loci crossing the calculated threshold for significance, but many small-effect QTLs underlying the complex traits remain undetected. The approach to calculation of correlations between marker effects outlined by Ziyomo and Bernardo (2013) thus provides true estimate of correlation between the effects of genomic regions affecting the traits. In our study it was confirmed that the directions as well as the sizes of correlations are comparable between the genetic correlations calculated from variance-covariance matrix and rrBLUP marker effects.

Four generations of repeated intermating at the F_2_ stage of the IBM population have increased the observed numbers of recombinations and feasibly broken a tight linkage. Consequently, a close genetic association between the traits might result due to pleiotropy rather than linkage. Thus, in our study tight genetic correlations as well as colocalized QTLs on chromosome 1 for ET/(TR-ET) and grain yield would indicate pleiotropy. Moreover, MCIM revealed several pleiotropic QTLs on chromosomes 1 and 8. The joint−mapping approach offered the advantage of directly testing whether the two traits affected by a particular QTL and provided greater power to detect pleiotropy. Eventually, fine−scale mapping with additional markers and larger mapping populations is required to distinguish truly pleiotropic loci from tightly linked loci not controlled by the same underlying genes. The same is true for the two complement IBM *Syn10* population with ten generations of random mating which were intermated after the F_2_ (Liu et al., 2015).

The efficiency of indirect over the direct selection for grain yield was lower for all three ChlF traits in both scenarios. The higher detected efficiencies in moderate heat scenario were caused by the increase of the genetic correlations in this scenario, and the highest value of 60.9% of the efficiency of the direct selection with indirect selection through the PI_ABS_ was caused by both increased genetic correlation and relatively high heritability, although no significant pleiotropic loci were detected for PI_ABS_ and grain yield. As a multiparametric expression calculated from other parameters with low to moderate heritability and genetically correlated with yield, PI_ABS_ is expected to have higher heritability, and to be genetically correlated with yield. Despite the relative inefficiency of the indirect over direct selection, indirect selection is often used in selection for abiotic stress tolerance (Bernardo, 2010). Ziyomo and Bernardo (2013) used several drought-related traits for indirect selection in the IBMSyn4 biparental population, and obtained efficiencies ranging from 48% for grain moisture, to 104% for use of ASI as drought tolerance indicator. The authors also found that the performance in non-stressed environments is relatively efficient (78%) in selection for performance in stressed environments.

In this study, the rrBLUP models were set to compare the obtained efficiencies of indirect selection with the efficiencies of genomic selection, as the genomic selection is also a form of indirect selection with $h_{y}$ = 1, assuming no genotyping errors (Bernardo, 2010). The low efficiency of genomic selection was found in both scenarios although it was 21.1% higher in moderate heat scenario. The low relative efficiencies of genomic selection through environments in this same dataset were reported earlier (Galić, 2018). Low relative efficiencies of genomewide selection and small partition of variance explained by the detected QTLs can be attributed to the masking effects of the tester line. Namely, if the Iodent tester heterotic to both IBM parents is fixed for dominant alleles for grain yield, the masking effect of the segregating loci is present (Mihaljevic et al., 2005; Peng et al., 2013). Massman et al. (2013) also found that within the biparental populations with saturated genetic maps efficiencies of the genomic selection are very low. They attributed the small proportion of variance explained to the probable causes of poorly addressing the genotype × environment variance, as well as the QTL × genetic background interaction. In our study, however, the genotype × environment variances were not significant. Although it was found in this study that genomewide predictions and indirect selection through the ChlF traits both display some predictive ability of grain yield, the aims of the two methods are different but complementary. The main aim of the genomic selection is to predict the performance of the untested progenies, thus saving the time and space for conducting the experiments, allowing for more efficient resource allocation in a breeding program. On the other hand, the main proposed aim of the indirect selection for grain yield through the ChlF parameters is more precise identification of the heat-stress tolerant progenies already included in breeding trials, or as part of the training populations. As the ChlF measurements can be conducted during the whole growing season, additional information may be captured. However, the aim of this study was to test the physiological effects of the heat during pollination when plants are the most sensitive to temperature extremes (Hatfield and Prueger, 2015) and to use this data in indirect selection for grain yield. Heat stress during the flowering time in terms of high night temperatures accompanied with high daytime temperatures was shown to cause the decrease in allelic effects of loci affecting the grain yield in moderate conditions (Millet et al., 2016). Furthermore, there is a completely heat-scenario dependent set of loci affecting the grain yield in environments with high night temperatures. Nevertheless, the genomic selection and ChlF approaches can be considered complementary rather than mutually exclusive, as there is a growing need for new and precise high throughput phenotyping strategies in genomics era (Cobb et al., 2013; Fiorani and Schurr, 2013). Also, there is a need for improvement of the present state of precision of the crop growth models (Cooper et al., 2016).

Our results demonstrated that ChlF via JIP-test is an appropriate method for realizing real-time, non-destructive monitoring of maize performance during flowering under moderate heat stress in the field environments. From an agronomical point of view, it seems that ChlF parameters may be used for predicting grain yield when heat stress occurred during vegetative and reproductive growth stages suggesting possible inclusion of the parameters in crop growth models. From a plant breeding point of view, using ChlF parameters ET/(TR-ET) and PI_ABS_ as secondary traits for selection under moderate heat stress could be an efficient breeding strategy for heat tolerance The ChlF parameters prove to be genetically correlated with grain yield in the stressed environments; they are genetically variable and not associated with grain yield under non-stressed conditions. Additionally, a breeding program for heat stress tolerance may be optimized by examining ChlF prior to yield trials in a pre-selected set of progenies harboring the heat-stress tolerance related alleles. Further studies on other breeding materials and linkage/association mapping are necessary to validate the presented QTLs, as well as to detect the additional loci associated with ChlF parameters in different heat scenarios. Specifically, future quantitative genetic studies on more severe (extreme) heat stress scenario would be worthwhile to examine changes in genetic correlations between ChlF parameters and grain yield. Eventually, the converging approaches of crop physiology, modeling, quantitative genetics and genomic prediction promise to considerably advance crop breeding for complex traits including adaptation to stress (Cabrera-Bosquet et al., 2012; Messina et al., 2018).

## Author Contributions

DS conceived and designed the experiments. AJ created and generated the testcross material. VG, MF, TL, AB, and ZZ organized and performed the experiments and collected the data. VG, MF, TL, and DS analyzed the data. VG and DS wrote the manuscript. All authors read and approved the final version of the manuscript.

## Conflict of Interest Statement

The authors declare that the research was conducted in the absence of any commercial or financial relationships that could be construed as a potential conflict of interest.

## Abbreviations

BLUPs: best linear unbiased predictions
ChlF: chlorophyll fluorescence
ET/(TR-ET): electron transport beyond $Q_{A}^{-}$
*F*_v_/*F*_m_: maximum quantum yield of PSII
ICIM: inclusive composite interval mapping
IRILs: intermated recombinant inbred lines
LOD: logarithm of odds
PI_ABS_: performance index (potential) for energy conservation from photons absorbed by PSII to the reduction of intersystem electron acceptors
PSII: Photosystem II
$Q_{A}^{-}$: quinone A
QTLs: quantitative trait loci
RC/ABS: reaction centers involved in $Q_{A}^{-}$ reduction per Photosystem II antenna chlorophyll
REML: restricted maximum likelihood
TR/DI: maximum quantum yield of primary photochemistry
VPD: vapor pressure deficit
