# Supplementary material for: Genetic Correlations Between Photosynthetic and Yield Performance in Maize Are Different Under Two Heat Scenarios During Flowering
Source: Front Plant Sci. 2019 Apr 30;10:566. doi: 10.3389/fpls.2019.00566 (PMC6503818; doi:10.3389/fpls.2019.00566)
Supplement: Supplementary file 1 [file Data_Sheet_1.PDF]

frontiers

Supplementary Material

Genetic Correlations between Photosynthetic and Yield Performance in Maize Are Different under Two Heat Scenarios during Flowering

Vlatko Galić, Mario Franić, Antun Jambrović, Tatjana Ledenčan, Andrija Brkić, Zvonimir Zdunić, and Domagoj Šimić*

*** Correspondence:** Domagoj Šimić: domagoj.simic@poljinos.hr

1 Supplementary Table 1. Mean values for three chlorophyll *a* fluorescence parameters[a] (RC/ABS, ET/(TR-ET), PI_{ABS}) measured during flowering in ear-leaf and grain yield (t/ha) in testcrosses of 221 intermated recombinant inbred lines (IRILs) of the IBM *Syn4* maize population grown in **Osijek (OS), Croatia** in 2014, 2015 and 2016.

	**OS14**				**OS15**				**OS16**			
**IRIL**	RC/ABS	ET/(TR-ET)	PI_{ABS}	Yield	RC/ABS	ET/(TR-ET)	PI_{ABS}	Yield	RC/ABS	ET/(TR-ET)	PI_{ABS}	Yield
M0001	0.42	2.86	3.86	14.51	0.40	2.67	3.97	10.99	0.53	1.68	4.30	10.82
M0003	0.38	3.01	5.94	11.56	0.52	2.43	5.47	11.56	0.54	1.60	4.27	6.41
M0005	0.48	3.01	6.13	10.09	0.52	2.64	5.89	11.06	0.53	2.08	4.95	8.70
M0007	0.50	2.89	4.05	13.46	0.50	2.60	5.54	11.73	0.52	1.95	4.64	9.27
M0008	0.49	2.86	3.70	14.75	0.49	2.25	4.92	11.02	0.49	1.87	4.12	11.28
M0010	0.40	2.44	5.33	9.44	0.50	2.15	5.17	9.19	0.46	3.07	5.33	7.54
M0011	0.50	2.92	4.22	12.64	0.54	2.64	6.46	10.63	0.54	2.35	5.07	6.07
M0012	0.48	2.57	5.13	13.44	0.51	2.58	5.91	12.29	0.55	1.90	4.85	4.39
M0013	0.43	2.81	5.23	11.30	0.50	2.09	4.59	9.43	0.52	2.06	4.93	6.32
M0014	0.45	2.46	4.33	11.03	0.46	3.07	5.46	12.80	0.56	1.99	5.21	10.46
M0015	0.43	2.76	4.16	14.14	0.50	2.19	4.92	10.27	0.55	1.77	4.70	9.57
M0016	0.48	2.53	4.77	12.51	0.52	2.47	5.74	12.36	0.46	2.95	5.43	6.07
M0017	0.45	2.51	3.38	13.71	0.49	2.51	5.59	10.50	0.52	2.00	4.62	9.07
M0019	0.44	2.90	3.63	10.18	0.52	2.44	5.86	13.32	0.55	2.05	5.07	4.01
M0021	0.36	2.96	3.38	11.95	0.52	2.13	5.19	11.37	0.52	1.97	4.55	8.28
M0022	0.50	2.69	2.47	11.66	0.48	2.10	4.72	8.05	0.53	1.55	3.79	11.85
M0023	0.46	2.52	3.85	12.20	0.57	2.46	6.85	9.94	0.57	2.91	6.87	8.77
M0024	0.45	2.88	2.78	10.91	0.52	2.56	6.22	8.41	0.51	1.88	4.37	8.35
M0025	0.45	2.90	3.07	13.36	0.42	2.28	3.80	16.08	0.52	1.88	4.36	10.40
M0026	0.44	2.09	3.54	15.35	0.48	2.03	4.65	11.53	0.44	1.92	4.03	7.33
M0027	0.43	2.73	3.88	12.30	0.49	2.53	5.17	15.85	0.41	1.86	4.69	12.93
M0028	0.38	2.75	2.05	11.03	0.45	1.48	2.81	14.92	0.49	2.07	4.46	7.85
M0029	0.49	2.50	3.13	13.06	0.49	2.27	4.90	11.54	0.50	1.95	4.17	6.42
M0031	0.49	2.51	3.34	16.20	0.49	2.74	5.67	9.43	0.49	1.87	3.97	10.06
M0032	0.50	2.94	4.70	13.74	0.49	2.37	5.22	10.38	0.50	1.99	4.48	6.41
M0033	0.66	2.44	4.99	13.70	0.49	1.91	4.82	16.17	0.52	2.03	4.84	8.95
M0034	0.46	2.65	4.29	12.63	0.47	1.95	4.39	11.81	0.50	3.44	6.56	11.11
M0035	0.49	2.79	3.69	10.89	0.53	2.68	6.23	12.19	0.52	2.27	5.21	10.18
M0036	0.46	2.61	3.58	16.08	0.47	2.62	5.09	13.06	0.49	2.73	4.01	10.96
M0039	0.49	2.52	5.38	14.94	0.49	2.23	5.09	12.14	0.52	2.35	5.29	6.58
M0040	0.43	3.30	4.70	5.88	0.39	1.96	3.02	4.53	0.52	1.91	4.40	6.47
M0041	0.44	2.56	5.25	13.62	0.46	2.11	4.51	10.21	0.50	2.04	4.36	9.97
M0043	0.52	2.53	3.91	11.91	0.50	2.43	5.29	9.70	0.54	1.96	4.81	10.81
M0044	0.43	2.40	3.59	13.16	0.46	1.83	3.86	11.67	0.54	1.85	4.75	10.18
M0047	0.44	2.48	4.33	7.54	0.43	1.34	4.06	7.77	0.52	1.89	4.45	8.11
M0048	0.34	1.75	1.46	13.69	0.47	2.54	4.78	11.53	0.52	1.71	4.01	9.06
M0051	0.46	2.54	3.06	12.82	0.49	2.67	5.56	10.44	0.53	1.75	4.35	8.31

M0052	0.47	2.39	2.65	10.44	0.49	2.34	5.12	10.18	0.52	1.82	3.25	4.36
M0054	0.46	3.05	2.10	9.68	0.52	2.33	5.38	10.53	0.54	1.95	4.88	8.78
M0055	0.43	2.67	3.58	11.36	0.51	2.32	5.58	7.45	0.56	2.09	5.23	8.79
M0056	0.36	1.39	1.55	12.63	0.46	2.27	4.54	11.79	0.50	2.10	4.50	7.75
M0057	0.49	2.93	4.88	13.75	0.44	2.49	4.43	13.45	0.53	2.17	4.98	10.31
M0058	0.44	2.48	3.51	13.50	0.51	2.35	5.24	9.63	0.49	2.04	4.12	10.28
M0059	0.47	3.03	4.52	11.43	0.44	2.38	4.51	12.45	0.49	2.18	4.31	9.57
M0060	0.48	2.52	3.34	11.64	0.50	2.21	5.40	12.19	0.46	2.00	3.74	8.07
M0061	0.57	2.29	4.27	7.80	0.73	2.97	7.36	7.30	0.50	2.06	4.31	9.41
M0063	0.47	2.08	3.58	13.26	0.49	2.25	4.95	13.45	0.58	1.88	5.00	6.76
M0066	0.46	2.39	4.52	11.06	0.54	2.19	5.73	12.72	0.44	2.01	3.53	8.75
M0068	0.46	2.60	4.44	10.97	0.52	2.61	6.13	10.99	0.49	1.72	3.53	9.49
M0071	0.46	2.38	4.12	12.65	0.49	2.29	5.17	11.32	0.50	2.03	4.35	11.04
M0075	0.39	2.26	4.66	13.72	0.51	2.57	5.80	10.73	0.50	1.86	4.09	10.49
M0077	0.43	2.59	3.85	8.65	0.49	2.06	5.02	10.94	0.52	2.10	4.79	9.25
M0080	0.49	2.79	3.75	13.16	0.45	1.88	3.50	13.69	0.51	1.73	4.47	8.62
M0081	0.47	2.92	4.39	13.88	0.52	2.36	5.62	9.97	0.52	1.90	4.28	10.56
M0082	0.44	2.56	4.08	12.38	0.45	2.53	4.30	11.88	0.45	3.23	5.40	8.20
M0083	0.50	2.66	3.74	12.74	0.59	2.68	6.87	10.11	0.51	2.07	4.60	8.33
M0085	0.38	1.93	3.37	9.77	0.54	2.45	6.27	8.90	0.53	2.34	5.46	3.45
M0086	0.45	2.38	3.85	11.94	0.47	2.26	4.59	9.71	0.52	1.98	4.42	8.13
M0087	0.48	2.26	3.63	11.91	0.45	1.82	5.06	12.74	0.50	2.34	5.06	10.18
M0090	0.48	2.46	3.99	11.78	0.49	2.19	4.76	8.13	0.54	2.17	5.03	5.52
M0091	0.47	2.44	4.54	13.06	0.52	2.90	4.52	11.13	0.54	1.80	4.29	8.56
M0092	0.43	2.45	4.30	12.44	0.51	1.92	4.46	10.62	0.52	2.22	4.98	7.75
M0093	0.54	2.13	4.36	12.08	0.59	2.99	5.65	13.95	0.49	2.18	4.27	10.02
M0096	0.41	2.63	4.45	10.75	0.53	2.00	5.23	7.77	0.42	2.17	4.02	6.92
M0097	0.41	2.31	4.26	11.55	0.47	2.01	4.18	11.99	0.43	1.96	3.33	10.07
M0098	0.43	2.66	4.89	11.45	0.50	2.06	4.73	12.12	0.50	1.89	3.97	10.08
M0099	0.47	2.58	3.83	9.87	0.44	1.90	3.48	9.22	0.48	2.17	4.21	8.26
M0106	0.39	2.33	4.51	11.75	0.44	2.23	5.06	9.39	0.46	2.02	3.62	9.60
M0109	0.50	2.84	4.15	12.64	0.49	2.59	5.50	12.65	0.51	2.07	4.53	11.22
M0110	0.41	2.99	4.32	11.47	0.51	2.11	4.97	11.11	0.38	1.77	3.99	10.91
M0111	0.40	2.74	5.20	12.13	0.53	2.60	6.18	11.46	0.48	2.09	4.25	7.88
M0114	0.39	2.68	4.60	9.34	0.53	2.10	5.48	11.24	0.47	2.10	4.00	8.34
M0116	0.52	2.48	4.02	13.84	0.52	2.09	5.14	9.39	0.50	2.07	4.56	9.55
M0118	0.45	2.39	4.37	13.25	0.47	2.88	5.38	12.50	0.51	2.24	5.11	8.17
M0119	0.39	2.38	3.52	9.85	0.49	2.14	4.71	9.08	0.54	2.54	5.87	10.34
M0121	0.40	2.82	4.38	12.95	0.56	2.11	5.11	8.17	0.49	1.88	3.69	8.80
M0123	0.47	2.29	4.24	9.16	0.66	2.51	7.36	10.25	0.48	2.13	4.24	8.31
M0124	0.49	2.63	4.89	9.33	0.53	2.01	5.16	11.11	0.47	1.98	3.62	5.09
M0125	0.48	2.46	3.56	11.80	0.51	2.00	4.69	13.47	0.52	2.08	4.82	7.98
M0126	0.46	2.48	3.78	11.33	0.52	2.52	5.79	11.11	0.50	2.01	4.23	11.72
M0127	0.43	2.40	2.35	13.76	0.50	2.06	4.99	11.55	0.50	1.97	4.20	9.06
M0128	0.56	2.54	4.57	7.59	0.50	1.98	4.83	9.77	0.49	2.21	4.49	10.01
M0130	0.45	2.42	4.32	12.01	0.50	2.34	4.90	8.99	0.44	1.72	3.96	10.55
M0131	0.40	2.16	4.10	12.06	0.54	2.69	6.65	7.95	0.49	2.14	4.31	9.87
M0133	0.46	2.59	5.07	10.48	0.48	2.76	5.55	10.82	0.49	2.07	4.17	10.27
M0143	0.39	2.65	4.34	12.23	0.51	1.97	4.72	12.97	0.47	2.10	3.91	10.06
M0145	0.42	2.17	4.35	13.99	0.47	2.01	4.29	10.64	0.54	2.63	5.64	14.33
M0146	0.45	2.35	3.00	13.16	0.52	2.37	5.43	14.36	0.48	2.01	4.11	4.18
M0150	0.41	2.99	4.10	10.55	0.49	2.74	4.94	11.31	0.46	2.19	4.17	10.11
M0151	0.46	2.34	3.73	13.42	0.52	2.82	5.50	12.01	0.46	2.01	3.77	9.76
M0152	0.55	2.79	3.24	11.26	0.49	2.02	4.49	12.62	0.47	1.82	2.95	9.13
M0153	0.55	2.73	3.71	13.26	0.46	2.21	5.25	8.92	0.47	2.35	4.58	8.11
M0154	0.56	2.79	4.71	14.69	0.49	2.08	4.73	9.64	0.48	2.34	4.78	10.89
M0157	0.50	2.59	3.97	12.19	0.59	2.95	6.29	10.21	0.49	2.46	4.94	9.56
M0160	0.50	2.47	4.22	13.71	0.46	1.90	4.40	15.44	0.48	2.39	4.63	9.37
M0161	0.50	3.13	4.36	13.95	0.57	2.64	5.26	11.03	0.48	2.28	4.56	11.46
M0162	0.52	2.69	4.76	13.25	0.49	2.03	4.29	9.61	0.48	2.17	4.21	10.36
M0163	0.39	2.16	2.32	9.45	0.55	3.07	7.03	14.33	0.47	2.29	4.39	9.49
M0165	0.49	2.62	3.58	16.17	0.50	2.48	5.45	10.33	0.52	2.53	5.57	12.28
M0167	0.50	2.81	3.11	13.26	0.54	2.22	5.84	8.95	0.48	2.11	4.08	7.72

M0168	0.50	2.78	4.44	12.83	0.81	2.72	8.60	9.40	0.51	2.22	4.96	9.47
M0169	0.52	2.69	2.78	13.28	0.51	2.59	5.81	7.59	0.50	2.42	5.25	8.32
M0171	0.50	2.75	3.58	14.04	0.53	2.34	5.71	9.65	0.50	2.25	4.86	8.77
M0172	0.59	2.70	6.47	11.43	0.55	3.21	5.99	15.35	0.50	2.57	5.50	10.69
M0174	0.53	2.92	5.13	16.08	0.55	2.74	6.13	11.59	0.53	2.47	5.62	10.63
M0176	0.50	2.27	3.76	7.35	0.57	3.44	8.60	5.44	0.50	2.42	4.78	6.34
M0177	0.51	2.63	5.03	16.11	0.49	2.45	5.26	12.44	0.47	2.38	4.70	11.51
M0180	0.50	2.68	4.37	13.01	0.48	1.89	4.27	11.98	0.51	2.11	4.52	1.79
M0181	0.49	2.70	4.55	10.49	0.49	2.61	5.45	8.46	0.50	3.44	6.85	11.69
M0182	0.52	2.68	4.33	14.93	0.53	2.54	6.27	10.09	0.48	2.09	4.08	9.83
M0185	0.51	2.62	5.37	13.82	0.49	2.54	5.39	9.87	0.49	2.30	4.86	10.03
M0186	0.53	2.49	4.75	11.73	0.52	2.41	5.45	10.47	0.53	2.34	5.53	10.53
M0187	0.51	2.81	4.23	13.53	0.45	2.36	4.04	13.14	0.47	2.14	4.23	9.74
M0188	0.48	2.68	4.44	10.42	0.58	2.40	5.71	9.34	0.52	2.39	5.33	10.02
M0189	0.53	2.61	4.34	14.37	0.55	2.37	6.03	13.80	0.40	1.67	1.95	8.93
M0190	0.54	2.37	4.86	13.22	0.49	1.80	4.27	9.44	0.50	2.19	4.65	7.93
M0191	0.45	2.46	4.68	12.15	0.55	2.48	6.27	13.25	0.51	2.20	4.85	9.85
M0194	0.54	2.81	4.18	9.05	0.55	2.61	6.31	9.04	0.52	2.20	3.71	6.18
M0195	0.51	2.74	4.33	11.47	0.44	1.77	4.09	8.50	0.48	2.32	4.63	8.32
M0197	0.50	2.45	4.50	13.63	0.50	2.15	4.87	10.11	0.54	2.40	5.59	8.15
M0199	0.50	2.72	3.93	12.61	0.52	2.50	5.98	9.14	0.51	2.39	5.33	7.07
M0200	0.49	2.91	4.70	14.55	0.51	2.55	5.65	11.16	0.53	2.12	4.77	11.00
M0201	0.58	3.01	5.99	9.51	0.44	1.55	4.94	5.30	0.52	2.41	5.46	6.36
M0205	0.50	2.78	4.41	9.93	0.51	2.44	5.55	11.27	0.52	2.51	5.64	8.14
M0206	0.35	2.57	3.52	11.13	0.53	2.91	5.89	5.44	0.53	2.57	6.01	9.54
M0208	0.57	3.44	5.94	12.08	0.59	3.01	5.92	14.77	0.50	2.50	5.33	10.67
M0209	0.49	2.81	3.53	14.49	0.53	2.15	5.19	10.21	0.50	2.22	4.84	10.14
M0210	0.50	2.87	3.27	11.36	0.51	2.16	5.42	10.38	0.52	2.37	5.25	9.85
M0213	0.47	1.64	4.05	12.74	0.53	2.41	5.87	12.31	0.47	2.14	4.19	11.09
M0214	0.38	2.54	2.30	7.78	0.50	2.13	5.17	9.08	0.50	2.38	4.82	9.45
M0215	0.49	2.78	4.94	14.08	0.50	2.50	5.52	12.05	0.49	2.37	4.82	6.07
M0216	0.48	2.51	4.08	13.30	0.48	2.74	5.68	10.94	0.46	2.00	3.76	7.82
M0218	0.50	2.36	4.07	14.38	0.53	2.13	5.19	10.34	0.51	2.17	4.66	10.70
M0219	0.39	1.55	2.30	7.41	0.54	2.37	5.13	5.94	0.51	2.20	4.94	8.21
M0220	0.45	2.21	3.41	13.20	0.51	2.99	5.20	9.39	0.52	2.02	4.70	9.26
M0222	0.50	2.50	3.95	11.69	0.50	1.95	4.62	12.58	0.48	2.34	4.70	11.58
M0224	0.49	2.99	4.58	11.39	0.52	2.15	5.30	9.73	0.52	2.31	5.18	10.04
M0228	0.37	1.86	1.69	5.97	0.49	3.21	6.36	8.02	0.49	2.32	4.88	6.43
M0230	0.52	2.39	3.75	12.16	0.46	1.48	3.36	7.99	0.50	2.15	4.78	7.91
M0233	0.52	2.80	3.48	13.09	0.40	2.39	3.42	11.74	0.48	2.42	4.94	9.87
M0236	0.50	2.34	3.52	12.91	0.51	2.39	5.52	10.69	0.47	2.16	4.40	9.47
M0240	0.52	2.51	4.36	11.80	0.44	2.03	4.95	11.57	0.47	2.32	4.29	8.16
M0241	0.52	2.94	4.89	11.75	0.49	2.38	5.00	10.62	0.50	2.63	5.44	7.41
M0248	0.52	2.78	3.60	12.38	0.52	2.48	5.86	12.33	0.47	2.55	5.04	7.51
M0253	0.51	2.74	4.24	12.96	0.50	2.33	5.70	10.48	0.63	2.36	5.59	6.76
M0255	0.55	3.05	3.61	9.56	0.55	2.16	5.76	10.72	0.48	2.37	4.79	8.72
M0258	0.49	2.72	2.77	14.05	0.47	2.27	4.88	9.73	0.47	2.52	5.03	9.09
M0262	0.48	2.62	4.63	12.62	0.46	2.21	4.47	13.56	0.51	2.40	5.34	9.55
M0263	0.53	2.83	3.89	12.42	0.38	1.88	2.44	11.73	0.51	2.57	5.71	11.42
M0264	0.50	2.53	3.63	13.69	0.53	2.27	5.66	10.37	0.50	2.39	5.26	8.69
M0265	0.42	2.97	3.81	10.86	0.49	1.90	4.16	11.94	0.48	1.93	4.39	9.42
M0266	0.46	2.57	3.27	13.22	0.51	2.30	5.50	12.61	0.44	1.86	3.34	10.61
M0267	0.54	2.61	4.01	9.70	0.56	2.45	6.38	10.00	0.49	2.54	5.06	5.18
M0268	0.54	2.99	5.93	11.69	0.42	1.58	4.03	7.02	0.49	2.47	5.27	7.60
M0269	0.43	2.44	3.13	7.76	0.49	3.01	7.27	13.16	0.52	2.57	5.80	9.77
M0270	0.49	2.19	4.18	12.38	0.52	1.95	4.58	11.47	0.47	2.36	4.43	11.74
M0272	0.51	2.86	3.25	12.96	0.55	2.46	5.63	8.51	0.51	2.62	5.68	7.73
M0274	0.55	2.77	2.02	10.59	0.52	2.19	5.35	11.19	0.51	2.58	5.75	7.94
M0275	0.46	2.92	2.15	14.79	0.46	2.30	4.26	10.79	0.49	2.37	4.95	9.19
M0276	0.51	2.55	2.15	12.61	0.51	2.77	5.88	10.59	0.49	2.38	5.06	6.74
M0278	0.53	2.78	3.07	11.45	0.51	2.16	5.27	8.58	0.45	2.46	4.60	6.18
M0280	0.50	2.53	2.07	10.72	0.49	2.24	4.85	12.31	0.51	2.50	5.55	8.23
M0282	0.46	1.83	2.00	11.69	0.48	2.35	5.99	13.49	0.46	2.33	4.47	7.85
M0283	0.54	2.72	2.73	11.93	0.51	2.54	5.92	9.34	0.50	2.52	5.40	8.16

M0284	0.49	2.58	1.37	12.41	0.52	2.59	5.63	10.45	0.50	2.44	5.28	5.74
M0287	0.53	2.60	5.38	11.56	0.55	2.64	5.94	9.67	0.54	2.82	6.77	9.21
M0288	0.53	2.66	1.27	14.00	0.48	2.15	4.66	12.11	0.49	2.41	5.00	10.28
M0289	0.47	2.53	3.52	13.13	0.44	2.39	4.31	13.50	0.48	2.49	5.21	7.76
M0295	0.46	2.46	4.25	10.21	0.50	2.99	6.85	12.30	0.49	2.43	5.04	9.27
M0297	0.39	3.04	2.97	12.07	0.47	2.44	4.71	10.21	0.52	2.55	5.72	8.64
M0298	0.48	3.07	3.78	11.40	0.52	2.28	5.30	9.77	0.47	2.43	4.75	6.02
M0303	0.49	2.56	3.92	13.87	0.43	2.22	4.03	13.44	0.35	1.58	3.06	11.46
M0304	0.49	2.53	4.23	12.74	0.47	1.97	4.38	13.28	0.50	2.09	4.55	10.04
M0305	0.46	2.52	3.69	9.24	0.51	2.28	5.16	13.01	0.49	2.24	4.76	7.00
M0307	0.42	3.13	3.73	12.58	0.49	2.06	4.85	11.96	0.45	2.06	4.32	12.69
M0310	0.49	2.47	4.17	13.75	0.52	2.35	5.57	11.82	0.50	2.50	5.32	6.86
M0311	0.55	2.47	3.87	12.41	0.50	2.00	4.77	11.08	0.47	2.35	4.51	7.94
M0313	0.52	2.96	4.11	11.42	0.46	2.28	4.36	11.92	0.49	2.59	5.19	8.20
M0314	0.50	2.35	3.90	9.42	0.51	2.54	5.59	13.48	0.46	2.38	4.51	8.37
M0315	0.52	2.72	3.81	14.79	0.53	2.77	6.55	7.57	0.47	2.37	4.63	10.75
M0317	0.52	2.78	4.27	13.17	0.51	2.37	5.23	11.50	0.49	2.60	5.34	8.97
M0318	0.50	2.35	4.17	13.87	0.49	2.32	5.36	10.86	0.47	2.60	4.93	7.35
M0321	0.51	2.89	3.66	12.69	0.53	2.49	5.65	9.65	0.50	2.55	5.20	7.68
M0322	0.48	2.22	3.72	10.73	0.50	2.34	5.21	12.17	0.50	2.28	4.95	8.47
M0323	0.51	2.66	4.06	14.03	0.51	2.37	5.74	9.75	0.47	2.39	4.98	11.68
M0325	0.49	2.52	3.95	14.52	0.50	2.49	5.46	8.90	0.48	2.13	5.25	8.29
M0326	0.50	2.80	4.41	12.33	0.46	2.84	4.95	10.70	0.48	2.54	5.29	3.29
M0327	0.40	2.88	4.08	11.32	0.51	3.13	5.98	10.94	0.47	2.27	4.41	5.74
M0328	0.42	2.31	3.61	11.74	0.47	2.25	4.52	9.45	0.48	2.20	3.95	10.50
M0329	0.49	2.58	4.22	10.49	0.52	2.37	5.54	9.52	0.46	2.16	3.82	8.52
M0331	0.50	2.25	4.14	14.55	0.51	2.44	5.72	12.80	0.47	2.05	3.49	8.59
M0334	0.48	2.43	3.95	11.79	0.50	2.18	5.15	9.54	0.46	1.29	3.02	11.00
M0335	0.51	2.69	5.28	12.02	0.55	2.20	5.70	9.86	0.50	2.43	5.15	7.33
M0337	0.45	2.24	3.23	14.74	0.51	2.04	4.87	12.50	0.45	2.31	4.16	8.85
M0338	0.47	2.66	4.52	9.93	0.49	1.93	4.49	11.67	0.50	2.61	5.40	9.15
M0340	0.48	2.92	3.98	12.78	0.52	2.17	4.99	7.71	0.50	2.14	4.66	8.40
M0341	0.45	2.67	3.69	12.71	0.45	2.34	4.46	12.53	0.52	2.29	4.91	8.47
M0345	0.52	2.97	4.48	12.00	0.44	2.15	3.92	10.39	0.45	1.68	3.47	4.43
M0346	0.54	2.67	3.61	11.98	0.51	2.10	4.91	11.13	0.45	1.94	4.12	7.70
M0347	0.50	2.54	3.76	10.53	0.52	2.58	5.93	13.30	0.55	2.33	4.78	21.64
M0349	0.42	2.45	3.46	12.31	0.50	2.44	5.21	6.15	0.50	2.56	5.31	8.41
M0351	0.50	2.84	3.85	12.89	0.50	2.22	4.76	13.01	0.45	2.29	4.01	9.13
M0352	0.51	2.75	3.27	14.91	0.50	2.01	4.51	11.90	0.50	2.46	4.89	11.36
M0354	0.45	2.20	3.00	10.46	0.46	1.88	3.97	10.02	0.48	2.44	4.58	8.60
M0355	0.43	2.51	3.60	10.66	0.49	2.07	4.66	8.45	0.50	2.65	5.45	9.22
M0356	0.48	2.49	2.28	13.69	0.47	2.05	4.27	9.45	0.37	1.86	2.19	3.29
M0357	0.39	2.61	3.04	12.31	0.37	2.58	3.15	12.02	0.46	2.26	4.03	8.72
M0360	0.51	2.59	4.57	13.95	0.53	2.21	5.39	14.05	0.50	2.34	4.91	8.31
M0361	0.50	3.41	5.81	11.54	0.46	1.99	6.27	9.64	0.50	2.59	5.26	7.94
M0364	0.47	2.61	4.62	13.83	0.54	2.46	5.78	9.76	0.47	2.31	4.37	8.25
M0368	0.43	2.40	4.27	10.80	0.51	2.77	5.89	10.23	0.46	2.43	4.61	7.05
M0369	0.45	2.60	4.54	13.27	0.45	2.12	4.14	11.80	0.48	2.42	4.45	8.50
M0373	0.47	2.40	4.07	9.64	0.52	2.37	5.59	11.99	0.45	2.17	3.93	8.59
M0374	0.51	2.57	4.42	10.26	0.48	2.30	5.16	10.61	0.47	2.17	4.20	8.38
M0378	0.49	2.64	3.63	12.02	0.45	2.79	4.95	14.15	0.51	2.56	5.11	7.11
M0379	0.43	2.63	4.26	11.77	0.52	2.34	5.48	10.80	0.51	2.49	5.27	8.80
M0380	0.46	2.41	3.82	11.89	0.46	1.67	3.14	11.79	0.46	2.09	3.69	6.22
M0381	0.44	2.79	3.86	12.75	0.50	2.17	4.85	8.10	0.49	2.35	4.67	7.55
M0382	0.39	2.64	4.12	13.43	0.50	2.42	5.39	10.31	0.51	2.52	5.18	9.26
M0383	0.51	2.55	3.66	10.61	0.53	2.56	6.09	9.69	0.47	2.35	4.55	8.30
M0384	0.45	2.75	3.70	14.63	0.45	2.35	4.19	12.24	0.42	2.24	5.04	7.82

[a] RC/ABS = reaction centers involved in Q_A^- reduction per PSII antenna chlorophyll; ET/(TR-ET) = electron transport beyond Q_A^-; PI_{ABS} = performance index (potential) for energy conservation from photons absorbed by PSII to the reduction of intersystem electron acceptors.
