# Supplementary material for: Genetic Correlations Between Photosynthetic and Yield Performance in Maize Are Different Under Two Heat Scenarios During Flowering
Source: Front Plant Sci. 2019 Apr 30;10:566. doi: 10.3389/fpls.2019.00566 (PMC6503818; doi:10.3389/fpls.2019.00566)
Supplement: Supplementary file 2 [file Data_Sheet_2.PDF]

Supplementary Material

2 **Supplementary Table 2.** Mean values for three chlorophyll *a* fluorescence parameters[a] (RC/ABS, ET/(TR-ET), PI_{ABS}) measured during flowering in ear-leaf and grain yield (t/ha) in testcrosses of 221 intermated recombinant inbred lines (IRILs) of the IBM *Syn4* maize population grown in **Ayvalik (AY), Turkey** in 2014, 2015 and 2016.

	AY14				AY15				AY16			
IRIL	RC/ABS	ET/(TR-ET)	PI_{ABS}	Yield	RC/ABS	ET/(TR-ET)	PI_{ABS}	Yield	RC/ABS	ET/(TR-ET)	PI_{ABS}	Yield
M0001	0.56	2.37	3.86	4.11	0.51	2.84	5.97	7.15	0.41	1.55	2.67	8.46
M0003	0.73	2.81	5.94	6.21	0.50	2.22	5.09	6.55	0.44	2.03	3.69	8.94
M0005	0.63	2.84	6.13	2.31	0.50	2.76	5.92	6.35	0.44	1.97	3.41	5.97
M0007	0.56	2.28	4.05	5.69	0.47	2.59	4.75	5.88	0.49	1.95	3.92	6.06
M0008	0.53	2.21	3.70	4.24	0.51	2.55	5.93	7.72	0.40	1.87	2.58	9.16
M0010	0.65	2.61	5.33	5.68	0.43	2.65	4.70	9.73	0.39	2.11	3.27	8.62
M0011	0.53	2.04	4.22	5.51	0.58	2.69	7.03	11.18	0.51	2.30	5.20	10.10
M0012	0.55	2.41	5.13	6.29	0.53	2.14	5.19	9.15	0.55	2.14	4.78	8.30
M0013	0.57	2.72	5.23	5.54	0.52	2.46	5.62	7.58	0.42	1.93	3.33	8.37
M0014	0.51	2.04	4.33	6.42	0.53	2.34	5.72	10.89	0.42	1.82	3.29	11.49
M0015	0.56	2.48	4.16	3.31	0.40	3.18	4.83	12.62	0.35	2.09	2.02	8.26
M0016	0.57	2.83	4.77	2.82	0.53	3.07	6.56	9.14	0.48	1.98	3.52	8.95
M0017	0.45	2.11	3.38	3.39	0.57	2.65	6.77	11.54	0.46	2.15	3.64	6.02
M0019	0.51	2.43	3.63	3.20	0.56	2.50	6.34	11.01	0.41	1.86	2.76	6.38
M0021	0.48	2.23	3.38	2.46	0.49	2.53	5.34	13.80	0.42	1.89	3.10	7.18
M0022	0.43	1.68	2.47	2.29	0.52	2.60	6.23	13.89	0.46	2.02	3.81	11.53
M0023	0.52	2.20	3.85	3.71	0.54	2.46	6.32	11.59	0.54	3.44	5.94	12.78
M0024	0.48	2.06	2.78	0.85	0.53	2.76	6.47	12.77	0.43	2.74	4.47	7.26
M0025	0.47	2.06	3.07	4.85	0.52	2.74	6.59	12.43	0.45	1.90	3.53	7.34
M0026	0.54	2.48	3.54	4.02	0.49	2.44	5.43	11.13	0.54	2.10	4.28	7.70
M0027	0.52	2.16	3.88	1.60	0.51	2.28	5.43	13.33	0.49	2.04	5.25	8.04
M0028	0.43	1.98	2.05	3.55	0.42	2.44	4.14	12.70	0.47	2.02	3.97	8.02
M0029	0.45	1.89	3.13	3.79	0.51	2.40	5.29	10.36	0.46	2.02	3.86	7.95
M0031	0.45	1.73	3.34	5.86	0.39	3.23	4.65	10.79	0.41	2.16	3.12	6.88
M0032	0.47	2.33	4.70	5.89	0.51	2.84	6.71	12.04	0.48	1.96	4.13	10.50
M0033	0.59	3.44	6.33	7.94	0.59	2.25	5.99	13.35	0.47	1.90	3.87	7.58
M0034	0.47	2.33	4.29	5.41	0.51	2.52	5.52	8.48	0.39	1.87	2.82	10.80
M0035	0.48	2.02	3.69	3.62	0.39	2.74	3.63	12.16	0.38	2.47	3.21	7.73
M0036	0.48	1.96	3.58	8.57	0.44	2.67	4.27	14.47	0.49	2.56	4.39	11.92
M0039	0.52	2.33	5.38	5.67	0.51	2.21	4.54	11.82	0.48	2.02	4.13	9.88
M0040	0.53	2.44	5.62	3.85	0.66	2.46	5.74	3.79	0.45	1.91	3.59	7.00
M0041	0.54	2.41	5.25	3.34	0.45	2.99	5.23	14.85	0.46	1.83	3.49	7.30
M0043	0.48	2.24	3.91	4.14	0.48	2.77	4.91	9.64	0.33	2.39	2.57	8.88
M0044	0.46	1.68	3.59	4.20	0.48	3.32	6.33	12.15	0.45	1.95	3.48	7.63
M0047	0.44	2.48	5.23	2.43	0.48	1.84	4.30	5.38	0.43	1.90	3.48	7.73
M0048	0.39	3.30	3.85	7.17	0.40	2.09	3.60	9.37	0.35	2.46	2.65	8.23
M0051	0.45	1.71	3.06	3.70	0.51	2.70	5.30	12.89	0.45	1.76	3.32	9.22
M0052	0.45	1.66	2.65	1.60	0.42	2.55	4.28	5.75	0.34	1.84	1.34	6.65
M0054	0.40	1.63	2.10	2.15	0.47	2.72	4.99	12.47	0.39	1.53	2.30	7.83
M0055	0.48	1.82	3.58	4.12	0.51	2.39	5.68	11.82	0.44	1.96	2.91	8.62
M0056	0.42	1.71	3.40	6.06	0.34	1.29	1.31	7.54	0.45	1.93	3.90	10.30
M0057	0.52	2.29	4.88	5.35	0.53	2.47	5.89	10.89	0.46	2.13	3.92	8.02
M0058	0.48	1.98	3.51	4.59	0.50	3.01	6.45	14.52	0.33	2.06	1.97	12.13
M0059	0.50	2.46	4.52	7.47	0.49	2.63	5.23	11.85	0.47	2.11	4.16	11.41
M0060	0.46	2.02	3.34	3.80	0.48	2.55	5.17	12.26	0.41	2.12	3.32	9.09
M0061	0.54	2.68	7.27	7.23	0.55	2.31	5.51	7.68	0.51	2.10	4.52	10.21
M0063	0.59	2.35	3.61	7.08	0.46	1.96	3.73	7.21	0.47	2.07	4.19	8.17
M0066	0.52	2.33	4.52	5.11	0.54	2.24	5.78	11.28	0.45	1.86	3.78	10.45
M0068	0.49	2.14	4.44	4.53	0.38	3.44	4.72	14.87	0.45	1.94	3.61	10.53

M0071	0.50	2.12	4.12	4.63	0.54	2.38	6.07	10.05	0.50	2.16	4.47	11.04
M0075	0.50	2.32	4.66	4.27	0.48	2.51	5.29	12.14	0.51	2.35	5.12	10.81
M0077	0.49	2.17	3.85	3.91	0.47	2.33	4.30	13.00	0.47	1.93	4.08	8.74
M0080	0.46	1.97	3.75	2.23	0.48	2.80	5.79	11.99	0.47	1.83	4.29	10.11
M0081	0.51	2.25	4.39	2.82	0.51	2.18	4.86	13.12	0.46	1.87	3.84	7.54
M0082	0.52	2.15	4.08	2.68	0.44	2.72	4.75	7.78	0.44	2.08	2.87	8.56
M0083	0.50	2.30	3.74	5.29	0.52	2.50	6.22	11.48	0.42	2.53	3.74	8.40
M0085	0.46	1.80	3.37	3.41	0.41	3.18	4.99	10.88	0.40	1.77	2.61	8.42
M0086	0.47	2.19	4.47	4.56	0.53	2.40	5.83	10.10	0.46	1.74	3.40	6.94
M0087	0.45	2.37	3.98	5.74	0.39	1.93	2.57	9.13	0.48	1.77	3.56	8.86
M0090	0.48	2.10	3.99	4.81	0.55	2.66	6.56	14.14	0.44	1.95	3.46	9.98
M0091	0.54	2.70	4.85	7.90	0.63	2.38	5.69	14.00	0.39	2.12	3.03	7.11
M0092	0.51	2.21	4.30	4.74	0.51	2.80	6.35	10.90	0.38	1.66	2.24	7.42
M0093	0.55	2.06	5.23	8.84	0.49	1.87	3.69	10.21	0.45	1.97	3.32	12.71
M0096	0.49	2.34	4.45	5.64	0.53	2.55	6.17	12.93	0.48	1.96	4.49	7.82
M0097	0.51	2.04	4.26	5.03	0.48	2.86	6.50	14.77	0.48	1.95	3.95	8.87
M0098	0.52	2.38	4.89	7.29	0.47	2.56	4.63	8.11	0.43	1.79	3.39	8.98
M0099	0.47	2.06	3.83	5.00	0.51	2.52	6.11	11.79	0.42	1.68	3.02	10.72
M0106	0.49	2.16	4.51	8.68	0.52	2.88	6.60	13.08	0.34	2.70	2.77	7.88
M0109	0.52	2.08	4.15	4.91	0.52	2.67	6.74	16.06	0.49	2.15	4.37	6.47
M0110	0.49	1.99	4.32	5.75	0.45	3.02	5.10	13.20	0.44	1.82	4.73	8.58
M0111	0.48	2.46	5.20	7.48	0.52	2.74	6.53	15.76	0.42	2.34	3.59	6.91
M0114	0.52	2.66	4.60	8.23	0.52	2.36	5.81	13.51	0.46	1.96	3.93	9.80
M0116	0.45	2.28	4.02	7.66	0.50	2.55	5.63	12.37	0.46	2.08	4.05	9.02
M0118	0.46	2.35	4.37	7.88	0.47	2.75	4.97	12.72	0.46	1.99	3.96	9.67
M0119	0.45	1.92	3.52	5.00	0.53	2.49	5.86	12.57	0.48	2.55	4.24	6.64
M0121	0.45	2.12	4.38	6.80	0.53	2.30	5.24	14.45	0.48	1.96	4.16	12.02
M0123	0.47	2.35	4.24	9.23	0.56	2.49	6.44	11.91	0.47	1.84	3.72	8.02
M0124	0.46	2.43	4.89	5.69	0.53	2.27	5.65	12.67	0.49	2.18	4.47	4.60
M0125	0.45	2.07	3.56	6.40	0.51	2.46	5.75	12.32	0.47	2.10	4.07	7.32
M0126	0.43	2.18	4.60	6.96	0.51	2.30	5.32	9.34	0.50	1.99	4.22	8.93
M0127	0.46	1.29	2.35	6.65	0.48	2.38	4.94	12.57	0.43	1.70	3.02	13.64
M0128	0.49	1.91	4.47	4.54	0.54	2.17	4.77	6.41	0.50	2.21	4.56	7.21
M0130	0.45	2.29	4.32	8.54	0.51	2.71	6.18	14.43	0.51	3.41	6.18	10.48
M0131	0.44	2.29	4.10	3.23	0.52	2.46	5.64	12.07	0.49	1.99	4.55	9.42
M0133	0.47	2.48	5.07	6.57	0.51	2.22	5.12	10.33	0.47	1.86	3.86	11.71
M0143	0.46	2.09	4.34	6.64	0.48	2.27	5.27	12.23	0.41	1.81	2.95	9.02
M0145	0.45	2.39	4.35	9.30	0.51	2.62	5.74	14.98	0.56	2.33	4.44	10.00
M0146	0.44	1.87	3.00	7.33	0.50	2.43	5.59	13.74	0.28	2.69	2.19	7.33
M0150	0.50	2.79	4.67	5.23	0.36	1.60	1.88	6.74	0.37	2.14	2.76	10.49
M0151	0.52	2.19	4.57	6.86	0.42	1.80	3.02	10.28	0.48	1.77	3.76	9.05
M0152	0.46	1.93	3.24	4.56	0.48	2.26	5.19	13.76	0.42	1.71	3.89	12.82
M0153	0.44	1.91	3.71	7.11	0.39	2.91	4.28	10.55	0.49	2.36	4.57	11.23
M0154	0.49	2.19	4.71	6.04	0.52	2.55	5.90	12.57	0.47	1.94	4.18	9.97
M0157	0.46	1.86	3.97	8.07	0.48	2.30	4.94	12.79	0.50	2.07	4.61	9.11
M0160	0.47	2.10	4.22	7.74	0.53	2.45	5.47	13.49	0.46	1.76	3.49	8.62
M0161	0.59	3.13	6.85	11.40	0.41	1.55	3.60	10.11	0.49	1.82	4.28	9.70
M0162	0.46	2.24	4.76	6.41	0.50	2.42	5.48	12.42	0.49	2.10	4.16	10.11
M0163	0.42	1.67	3.10	4.38	0.39	1.82	4.00	10.18	0.53	2.33	4.84	7.54
M0165	0.43	1.85	3.58	6.11	0.54	2.43	6.27	14.35	0.50	2.05	4.27	8.32
M0167	0.45	2.06	3.11	4.75	0.51	2.63	6.01	13.77	0.50	1.98	4.31	9.34
M0168	0.47	1.86	4.44	4.87	0.50	2.56	5.60	10.87	0.42	2.04	3.28	7.21
M0169	0.42	1.58	2.78	1.60	0.51	2.72	5.72	13.76	0.50	1.92	4.24	10.18
M0171	0.44	1.98	3.58	6.03	0.50	2.69	5.98	16.18	0.48	1.78	3.87	9.97
M0172	0.47	1.99	4.56	8.54	0.50	1.96	4.60	10.74	0.52	2.13	4.77	10.67
M0174	0.51	1.90	3.78	7.48	0.43	1.77	4.78	11.59	0.53	2.08	4.90	12.36
M0176	0.52	2.10	4.51	0.85	0.40	1.50	2.95	6.86	0.55	2.09	5.36	9.11
M0177	0.48	2.31	5.03	6.22	0.50	2.34	5.67	14.77	0.51	2.84	5.69	9.53
M0180	0.47	2.15	4.37	8.31	0.45	2.21	3.99	13.35	0.51	1.98	4.76	13.96
M0181	0.49	2.33	4.55	5.52	0.50	2.22	4.85	10.80	0.47	3.44	6.23	11.04
M0182	0.49	1.98	4.33	5.36	0.51	2.72	6.22	11.47	0.46	1.85	3.78	11.31
M0185	0.50	2.43	5.37	7.62	0.48	2.31	4.78	10.09	0.44	1.73	3.41	9.83
M0186	0.50	2.28	4.75	5.77	0.50	2.54	5.73	9.15	0.38	2.55	3.19	12.19

M0187	0.47	1.96	4.23	5.85	0.48	2.53	5.13	13.64	0.48	1.91	4.01	7.55
M0188	0.47	2.29	4.44	9.99	0.49	2.72	5.95	13.14	0.47	2.13	4.28	11.75
M0189	0.47	1.85	4.34	9.24	0.46	2.73	4.58	13.60	0.59	2.44	4.48	7.73
M0190	0.50	2.36	4.86	5.22	0.51	2.56	5.73	10.91	0.50	2.03	4.20	8.26
M0191	0.46	2.44	4.68	6.86	0.54	2.46	5.88	11.22	0.48	2.08	3.94	7.64
M0194	0.46	1.88	4.18	8.07	0.43	3.30	4.26	12.03	0.54	2.43	5.51	12.58
M0195	0.49	2.27	4.33	8.11	0.50	2.20	5.21	13.06	0.46	1.97	4.02	8.35
M0197	0.46	2.00	4.50	5.45	0.52	2.18	5.07	12.26	0.48	1.92	4.12	7.64
M0199	0.46	1.92	3.93	6.59	0.48	2.37	4.59	12.50	0.48	2.07	4.21	11.15
M0200	0.48	2.18	4.70	6.66	0.48	2.29	4.91	9.66	0.51	2.36	5.07	6.02
M0201	0.66	2.97	7.27	5.49	0.44	1.82	4.56	5.75	0.47	2.19	4.21	6.27
M0205	0.47	1.98	4.41	5.49	0.51	2.16	5.10	8.12	0.49	2.07	4.26	8.02
M0206	0.57	2.99	6.39	4.91	0.41	2.30	4.35	9.73	0.41	2.37	3.68	7.35
M0208	0.44	1.24	3.99	9.78	0.52	2.55	5.36	13.35	0.45	1.81	3.64	8.54
M0209	0.46	2.16	3.53	4.68	0.47	2.27	4.65	11.25	0.40	1.55	2.86	6.76
M0210	0.46	1.86	3.27	6.66	0.48	2.96	5.73	8.78	0.46	1.76	3.62	8.56
M0213	0.47	1.95	4.05	7.17	0.48	2.12	4.21	7.44	0.45	1.87	3.50	10.94
M0214	0.47	2.08	3.99	10.50	0.49	2.15	4.57	10.44	0.42	1.84	3.24	8.17
M0215	0.48	2.32	4.94	5.07	0.49	2.08	4.58	13.00	0.42	1.99	3.49	9.26
M0216	0.46	1.97	4.08	4.54	0.43	2.13	3.52	7.20	0.39	1.88	2.91	8.00
M0218	0.49	2.24	4.07	5.56	0.45	1.88	3.60	8.03	0.44	2.06	3.52	6.96
M0219	0.51	2.03	4.61	3.34	0.48	2.08	3.32	2.43	0.43	1.91	3.40	7.78
M0220	0.42	1.67	3.41	6.81	0.47	2.13	4.25	8.59	0.49	1.88	3.96	5.84
M0222	0.46	2.29	3.95	3.68	0.43	2.00	3.68	10.09	0.47	1.91	4.03	8.06
M0224	0.46	1.85	4.58	6.56	0.42	1.52	2.47	9.89	0.43	2.06	3.45	10.34
M0228	0.47	2.52	4.17	4.47	0.37	1.69	2.50	2.15	0.44	1.95	3.40	0.00
M0230	0.44	1.99	3.75	7.67	0.43	2.19	3.18	13.66	0.47	1.99	3.77	8.71
M0233	0.44	1.77	3.48	5.17	0.43	2.79	4.62	11.11	0.40	1.90	2.64	7.08
M0236	0.44	1.95	3.52	6.99	0.46	1.89	4.06	10.24	0.39	1.84	2.90	8.61
M0240	0.46	1.76	4.36	7.41	0.39	3.30	4.66	8.84	0.50	2.10	4.76	10.02
M0241	0.45	2.12	4.89	7.44	0.45	2.09	4.09	13.60	0.47	1.97	3.81	7.34
M0248	0.45	1.93	3.60	6.34	0.46	1.88	3.81	10.28	0.46	2.01	4.12	6.90
M0253	0.48	1.82	4.24	7.79	0.49	2.27	4.33	10.58	0.43	2.06	4.27	6.93
M0255	0.45	1.73	3.61	8.15	0.45	1.97	3.72	9.00	0.41	2.02	3.19	9.82
M0258	0.42	1.67	2.77	5.09	0.42	2.08	3.69	9.41	0.43	2.03	3.63	8.02
M0262	0.48	1.95	4.63	6.17	0.46	2.09	4.23	9.64	0.39	1.65	2.32	6.45
M0263	0.43	1.77	3.89	6.36	0.47	2.44	4.94	11.85	0.37	2.01	2.63	8.24
M0264	0.45	1.58	3.63	5.80	0.48	2.60	5.40	12.16	0.35	1.84	1.74	8.56
M0265	0.44	2.08	3.81	6.62	0.47	2.31	4.65	7.36	0.43	1.98	3.93	8.75
M0266	0.41	1.84	3.27	9.36	0.45	1.99	3.73	8.53	0.45	2.02	3.85	10.34
M0267	0.46	1.97	4.01	2.67	0.48	1.92	4.15	7.22	0.48	2.04	3.93	4.78
M0268	0.43	2.02	3.78	1.60	0.44	1.80	4.30	8.56	0.47	2.02	3.70	8.56
M0269	0.39	1.92	3.92	9.35	0.37	2.14	3.48	7.55	0.38	1.66	2.43	4.82
M0270	0.48	2.16	4.18	4.74	0.44	2.01	3.35	6.53	0.44	1.95	3.15	4.75
M0272	0.42	1.87	3.25	3.60	0.46	2.54	4.86	6.62	0.44	2.26	3.55	8.56
M0274	0.39	1.59	2.02	4.47	0.47	2.85	5.25	7.23	0.54	2.16	4.89	6.87
M0275	0.40	1.74	2.15	2.79	0.48	2.37	4.91	6.93	0.47	2.21	4.04	9.42
M0276	0.37	1.31	2.15	1.98	0.44	2.70	4.70	9.77	0.50	1.92	4.04	7.82
M0278	0.43	1.71	3.07	3.49	0.47	1.97	4.06	9.69	0.44	1.98	3.76	5.89
M0280	0.39	1.49	2.07	3.07	0.37	3.05	3.95	10.88	0.46	1.86	3.54	4.60
M0282	0.38	1.30	2.00	3.46	0.46	2.30	4.36	13.93	0.51	2.08	4.62	7.31
M0283	0.43	1.99	2.73	3.34	0.49	2.32	5.05	9.54	0.43	1.76	3.11	7.48
M0284	0.36	1.27	1.37	2.87	0.49	2.25	4.88	9.78	0.48	2.25	3.72	9.35
M0287	0.56	2.68	5.63	5.64	0.50	2.33	4.51	8.75	0.48	1.88	3.86	6.86
M0288	0.36	1.33	1.27	2.43	0.48	2.00	4.54	9.36	0.42	1.77	2.99	8.32
M0289	0.44	1.87	3.52	6.80	0.46	2.34	4.43	10.80	0.46	2.12	4.00	6.97
M0295	0.52	2.59	5.70	5.17	0.37	1.86	3.48	4.09	0.44	1.82	3.39	6.79
M0297	0.41	1.81	2.97	4.88	0.44	1.96	3.65	9.57	0.49	2.13	4.48	7.01
M0298	0.43	1.93	3.78	5.89	0.46	1.66	3.22	6.25	0.42	1.73	3.40	7.23
M0303	0.46	1.82	3.92	4.43	0.46	2.17	4.22	6.64	0.44	2.09	3.58	10.03
M0304	0.45	1.93	4.23	9.97	0.45	2.03	3.90	8.52	0.45	1.99	3.76	9.13
M0305	0.44	1.76	3.69	7.06	0.50	2.61	5.58	7.70	0.47	1.84	3.84	2.59
M0307	0.42	1.77	3.73	5.56	0.45	2.45	4.62	9.34	0.41	2.39	3.63	7.35
M0310	0.44	2.04	4.17	5.73	0.47	2.25	4.46	8.58	0.44	2.12	3.56	6.91

M0311	0.45	1.81	3.87	6.56	0.50	2.68	5.54	12.30	0.42	1.82	3.14	3.90
M0313	0.45	1.91	4.11	4.36	0.46	1.87	3.63	7.90	0.42	1.73	2.72	4.93
M0314	0.44	1.82	3.90	6.70	0.43	1.60	2.95	8.01	0.40	1.91	2.67	6.17
M0315	0.42	1.67	3.81	8.19	0.44	2.02	3.76	7.04	0.45	1.88	3.63	7.54
M0317	0.43	1.99	4.27	4.91	0.45	1.88	3.50	6.36	0.44	1.94	3.48	9.01
M0318	0.43	1.77	4.17	6.87	0.39	1.58	2.29	7.24	0.43	2.15	3.62	6.36
M0321	0.42	1.79	3.66	2.90	0.42	1.52	2.56	4.50	0.50	1.89	4.00	6.74
M0322	0.44	1.94	3.72	5.38	0.39	1.34	2.06	4.91	0.43	1.99	3.63	6.08
M0323	0.45	1.94	4.06	7.20	0.44	1.54	2.82	6.18	0.47	2.05	4.33	9.06
M0325	0.45	1.78	3.95	8.52	0.47	1.91	3.63	6.31	0.46	1.88	4.89	9.13
M0326	0.45	1.96	4.41	6.06	0.47	1.95	3.83	4.55	0.47	1.97	4.04	8.26
M0327	0.43	2.29	4.27	6.29	0.43	2.19	5.13	11.95	0.44	1.99	3.60	6.54
M0328	0.42	1.87	3.61	5.93	0.47	1.96	4.09	7.57	0.46	1.90	3.77	8.08
M0329	0.46	2.13	4.22	7.56	0.50	2.04	4.22	5.37	0.53	2.21	3.68	2.82
M0331	0.44	2.05	4.14	8.56	0.45	1.65	3.08	5.74	0.46	2.19	3.98	8.80
M0334	0.47	1.83	3.95	6.74	0.40	1.49	2.09	5.40	0.52	2.29	3.65	7.22
M0335	0.43	2.39	5.28	6.40	0.43	1.52	2.56	7.21	0.49	2.17	4.35	7.89
M0337	0.43	1.80	3.23	5.10	0.48	1.84	3.47	6.37	0.46	1.78	3.68	6.91
M0338	0.45	2.01	4.52	8.60	0.39	1.52	1.70	4.08	0.49	2.22	4.21	5.74
M0340	0.45	1.82	3.98	7.11	0.50	2.08	4.59	6.43	0.46	1.89	3.55	6.54
M0341	0.42	1.69	3.69	7.53	0.50	2.18	4.49	6.69	0.41	1.93	3.28	7.48
M0345	0.42	2.10	4.48	7.07	0.47	2.23	4.08	6.52	0.52	2.69	4.85	6.13
M0346	0.43	1.64	3.61	5.84	0.48	1.92	4.00	4.40	0.42	1.73	2.07	7.45
M0347	0.42	1.68	3.76	4.84	0.50	2.05	4.52	5.96	0.46	1.95	4.20	11.74
M0349	0.42	1.80	3.46	4.38	0.50	2.10	4.57	7.02	0.43	1.71	3.07	5.06
M0351	0.42	1.71	3.85	4.53	0.50	2.29	5.14	6.88	0.38	1.87	2.60	8.82
M0352	0.40	1.81	3.27	6.85	0.49	2.02	4.51	6.41	0.43	1.75	3.00	4.09
M0354	0.43	1.53	3.00	4.21	0.49	2.19	4.60	6.46	0.41	1.74	2.96	4.48
M0355	0.42	1.86	3.60	2.96	0.46	2.16	4.01	5.89	0.41	2.10	3.16	5.68
M0356	0.40	1.54	2.28	6.19	0.46	1.81	3.29	6.05	0.43	2.32	4.06	10.21
M0357	0.41	1.93	3.04	4.33	0.50	2.27	5.11	5.76	0.44	1.83	3.23	4.84
M0360	0.44	1.99	4.57	6.70	0.53	2.59	6.12	8.51	0.46	2.23	3.66	7.11
M0361	0.44	1.80	5.43	7.82	0.52	2.56	5.94	6.41	0.46	2.12	4.10	7.46
M0364	0.43	1.85	4.62	7.40	0.51	2.47	5.71	8.92	0.51	2.25	4.79	6.98
M0368	0.43	1.89	4.27	3.39	0.53	2.51	5.76	8.83	0.47	2.01	3.76	5.68
M0369	0.43	1.69	4.54	7.52	0.48	1.99	4.39	9.85	0.45	2.20	3.57	5.53
M0373	0.43	1.71	4.07	6.60	0.50	2.41	5.28	9.53	0.48	2.26	4.25	6.14
M0374	0.43	1.80	4.42	7.45	0.52	2.11	5.27	8.70	0.48	2.41	4.56	8.97
M0378	0.44	1.56	3.63	6.44	0.54	2.32	5.61	7.36	0.47	1.97	3.62	6.89
M0379	0.46	1.96	4.26	6.96	0.51	2.23	4.99	7.96	0.46	2.00	3.70	9.78
M0380	0.43	1.66	3.82	5.94	0.48	1.93	3.79	8.73	0.44	1.85	3.23	8.29
M0381	0.43	1.76	3.86	6.28	0.43	1.48	2.75	7.85	0.48	2.16	4.45	6.50
M0382	0.42	1.98	4.12	8.60	0.51	2.34	5.07	7.80	0.44	2.21	3.95	7.01
M0383	0.41	1.75	3.66	7.46	0.52	2.34	5.46	8.30	0.49	2.18	4.45	7.77
M0384	0.40	1.67	3.70	3.83	0.50	2.25	5.28	9.07	0.50	2.36	6.95	9.77

[a] RC/ABS = reaction centers involved in Q_A^- reduction per PSII antenna chlorophyll; ET/(TR-ET) = electron transport beyond Q_A^-; PI_{ABS} = performance index (potential) for energy conservation from photons absorbed by PSII to the reduction of intersystem electron acceptors.
