# Supplementary material for: Genetic Correlations Between Photosynthetic and Yield Performance in Maize Are Different Under Two Heat Scenarios During Flowering
Source: Front Plant Sci. 2019 Apr 30;10:566. doi: 10.3389/fpls.2019.00566 (PMC6503818; doi:10.3389/fpls.2019.00566)
Supplement: Supplementary file 3 [file Data_Sheet_3.PDF]

frontiers

# *Supplementary Material*

3 **Supplementary Table 3.** Mean values ± standard error of the mean for three JIP-test parameters and grain yield in 221 testcrosses of the IRILs grown in six environments i.e. two locations of Osijek, Croatia (OS) and Ayvalik, Turkey (AY) classified into two heat scenarios.

| **Environment** | **RC/ABS**[a] | ***ET/(TR-ET)*** | **$PI_{ABS}$** | **Yield** |
|---|---|---|---|---|
| OS14 | 0.475 ± 0.003 | 2.60 ± 0.02 | 3.94 ± 0.06 | 12.15 ± 0.12 |
| OS15 | 0.501 ± 0.003 | 2.34 ± 0.02 | 5.21 ± 0.06 | 10.87 ± 0.13 |
| OS16 | 0.494 ± 0.002 | 2.23 ± 0.02 | 4.67 ± 0.05 | 8.79 ± 0.14 |
| AY14 | 0.468 ± 0.003 | 2.06 ± 0.02 | 4.06 ± 0.06 | 5.76 ± 0.15 |
| AY15 | 0.422 ± 0.004 | 1.91 ± 0.03 | 3.14 ± 0.09 | 9.28 ± 0.20 |
| AY16 | 0.454 ± 0.003 | 2.04 ± 0.02 | 3.77 ± 0.05 | 8.29 ± 0.14 |
| *Mild heat (Mean OS)* | 0.49 ± 0.001 | 2.39 ± 0.01 | 4.70 ± 0.03 | 10.95 ± 0.08 |
| *Moderate heat (Mean AY)* | 0.45 ± 0.002 | 2.08 ± 0.01 | 3.94 ± 0.04 | 8.33 ± 0.10 |

[a] RC/ABS = reaction centers involved in $Q_A^-$ reduction per Photosystem II antenna chlorophyll; ET/(TR-ET) = electron transport beyond $Q_A^-$; $PI_{ABS}$ = performance index (potential) for energy conservation from photons absorbed by PSII to the reduction of intersystem electron acceptors.
