# Supplementary material for: Genetic Correlations Between Photosynthetic and Yield Performance in Maize Are Different Under Two Heat Scenarios During Flowering
Source: Front Plant Sci. 2019 Apr 30;10:566. doi: 10.3389/fpls.2019.00566 (PMC6503818; doi:10.3389/fpls.2019.00566)
Supplement: Supplementary file 4 [file Image_1.PDF]

Supplementary Material

Genetic Correlations between Photosynthetic and Yield Performance in Maize Are Different under Two Heat Scenarios during Flowering

Vlatko Galić, Mario Franić, Antun Jambrović, Tatjana Ledenčan, Andrija Brkić, Zvonimir Zdunić, and Domagoj Šimić*

*** Correspondence:** Domagoj Šimić: domagoj.simic@poljinos.hr

3 Supplementary Figure S1

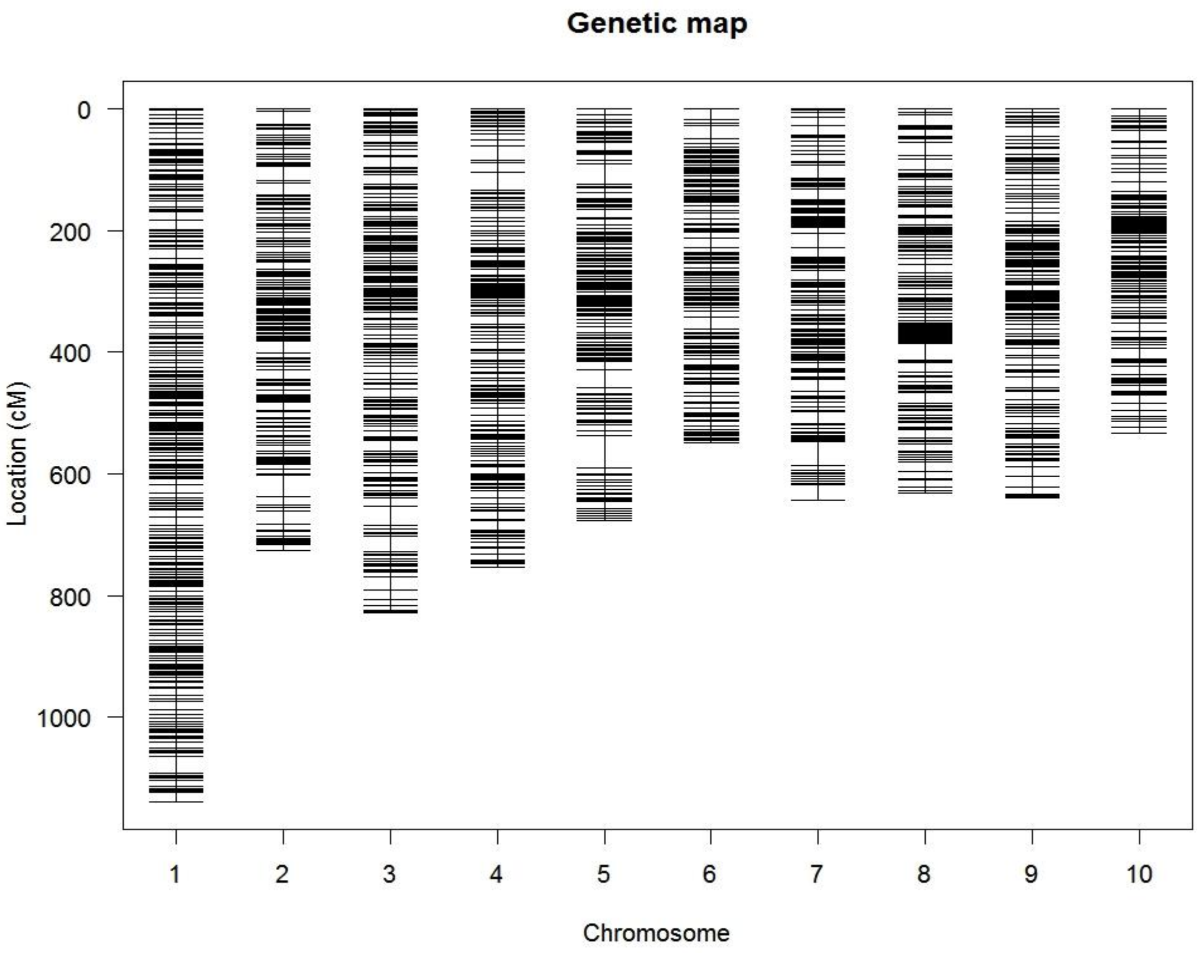

Supplementary Figure 1. Estimated genetic map of the IBM*Syn4* maize population. Horizontal lines on the chromosomes represent individual markers ($N_{MARKERS}$ = 2178) and their corresponding positions (y- axis).
